# Efficiency of different measures for defining the applicability domain of classification models

**DOI:** 10.1186/s13321-017-0230-2

**Published:** 2017-08-03

**Authors:** Waldemar Klingspohn, Miriam Mathea, Antonius ter Laak, Nikolaus Heinrich, Knut Baumann

**Affiliations:** 1 Institute of Medicinal and Pharmaceutical Chemistry, University of Technology Braunschweig, Beethovenstrasse 55, 38106 Brunswick, Germany; 2 Bayer Pharma Aktiengesellschaft, Computational Chemistry, Müllerstrasse 178, 13353 Berlin, Germany

**Keywords:** Applicability domain, Applicability domain measures, Reject option, Novelty detection, Confidence estimation, Class probability estimation

## Abstract

The goal of defining an applicability domain for a predictive classification model is to identify the region in chemical space where the model’s predictions are reliable. The boundary of the applicability domain is defined with the help of a measure that shall reflect the reliability of an individual prediction. Here, the available measures are differentiated into those that flag unusual objects and which are independent of the original classifier and those that use information of the trained classifier. The former set of techniques is referred to as novelty detection while the latter is designated as confidence estimation. A review of the available confidence estimators shows that most of these measures estimate the probability of class membership of the predicted objects which is inversely related to the error probability. Thus, class probability estimates are natural candidates for defining the applicability domain but were not comprehensively included in previous benchmark studies. The focus of the present study is to find the best measure for defining the applicability domain for a given binary classification technique and to determine the performance of novelty detection versus confidence estimation. Six different binary classification techniques in combination with ten data sets were studied to benchmark the various measures. The area under the receiver operating characteristic curve (AUC ROC) was employed as main benchmark criterion. It is shown that class probability estimates constantly perform best to differentiate between reliable and unreliable predictions. Previously proposed alternatives to class probability estimates do not perform better than the latter and are inferior in most cases. Interestingly, the impact of defining an applicability domain depends on the observed area under the receiver operator characteristic curve. That means that it depends on the level of difficulty of the classification problem (expressed as AUC ROC) and will be largest for intermediately difficult problems (range AUC ROC 0.7–0.9). In the ranking of classifiers, classification random forests performed best on average. Hence, classification random forests in combination with the respective class probability estimate are a good starting point for predictive binary chemoinformatic classifiers with applicability domain.

**Electronic supplementary material:**

The online version of this article (doi:10.1186/s13321-017-0230-2) contains supplementary material, which is available to authorized users.

**Graphical abstract:**
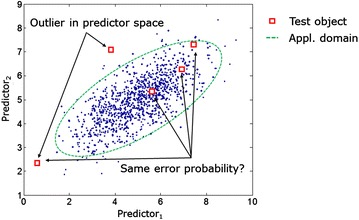
.

## Background

Classification rules are often used in chemoinformatics to predict categorical properties such as bioactivity, toxicity or metabolic stability of drug candidates. The classification rule is derived from *n* training set compounds where each chemical compound is represented by *p* explanatory variables (molecular descriptors) and a class label or property value [1, 2]. In the productive phase of the classifier, new objects (future objects) are predicted using only the information of their molecular descriptors [3, 4]. For decision making, e.g. for prioritizing the order of synthesis of candidate molecules, an important piece of information is the uncertainty associated with the prediction of a particular molecule. The prediction error, estimated with an independent test set, provides important information on the average performance of the employed classifier. However, it cannot provide information about the probability of misclassification for a particular molecule. There are two situations where the individual probability of misclassification may differ significantly from the average probability of misclassification (i.e. the prediction error). First, a future object may be dissimilar to the training objects in terms of its molecular descriptors. It is reasonable to expect a larger probability of misclassification for those molecules that are located in sparsely populated regions of the training data set. Second, a future object may be located close to the decision boundary of the classifier. In most real-world data sets class overlap is strongest in that region. This may be due to label noise (i.e. the feature that determines the class label can only be determined up to a certain precision) or due to imperfect molecular descriptors that cannot differentiate between subtle features of the classes. In any case, the user may wish to be informed about uncertain predictions. This is commonly done by defining an applicability domain (AD) in chemoinformatics. The latter is defined as the “response and chemical structure space in which the model makes predictions with a given reliability” [5]. Predictions for molecules located outside the AD are considered to be unreliable. An AD is one of the pillars of a validated model according to the OECD principles for quantitative structure–activity relationship (QSAR) models [6].

While it is pretty straightforward to define the requirements for an ideal AD, it is less clear how these requirements can be achieved. How can the response and chemical structure space be narrowed down so that only reliable predictions remain? In the first case mentioned above various distance measures have been employed as to characterize how well the future object is embedded in the training set [7–10]. If the future object is too remote from the training data set, its prediction is rejected. Remote objects typically contain novel concepts not represented in the training data set which increases the error probability. Identifying remote objects is often termed novelty detection, anomaly detection or outlier detection in machine learning [8, 11–14]. In the following, the term novelty detection is used. In novelty detection the training data set is used to define a region with known objects and any object that does not belong to this region is flagged as novel. Since only the class of normal objects is defined (i.e. the training set) while the class of novel objects is ill-defined, this is a so-called one-class classification problem and any one-class classifier can be used for novelty detection. It is important to note that the one-class classifier used for novelty detection does neither use the class label information of the objects in the training data set nor information of the underlying classifier that is used to predict the object’s class label. Novelty detection solely uses the explanatory variables to set up a second classifier to determine whether or not a future object is close enough to the known objects.

Remoteness to the training data certainly determines the reliability of a prediction. However, an even stronger predictor for the expected probability of misclassification should be an object’s distance to the decision boundary of the classifier. A small distance to the decision boundary was the second case mentioned above that may yield an error probability above average. Characterizing the probability of misclassification of an individual object has been termed confidence estimation [15, 16]. As opposed to novelty detection, confidence estimation uses information of the underlying classifier. Most confidence measures are built-in measures of the employed classifier that characterize, one way or the other, the distance of the future object to the decision boundary. This distance is then converted to a degree of class membership. These values can be strict probabilities such as the posterior probabilities in linear discriminant analysis or they can be uncalibrated scores. In this case, the only property that holds is that a higher score indicates a higher probability of class membership [17, 18]. There are techniques to convert uncalibrated scores into estimated class probabilities [19–21]. If calibrated properly, the class membership probability is related to the probability of misclassification for that object. Confidence estimators can also be derived from ensemble predictions by using the classifier stability to estimate a class membership score [22]. Ensemble members of a stable classifier always predict the same class for a particular object. An instable classifier varies in its predictions. The fraction of votes for one class can be used as a class membership score.

Many novelty and confidence measures have been explored for defining the AD [15]. These measures have been termed distance to model (DM) measures in chemoinformatics [9, 23]. A small distance to the underlying classification model implies reliable predictions. Since most of the employed measures are actually no distances, the umbrella term used here will simply be applicability domain measures to avoid misunderstandings. In accord with DM measures, increasing AD measures will also indicate a larger error probability for the respective prediction. Despite the fact that many AD measures have been explored, a larger benchmark study comparing these measures is still missing. There is one landmark study that compares many AD measures but it does so on just a single data set [9]. It has been noted in this study that AD measures based solely on explanatory variables (i.e. novelty detection) are less powerful for defining the AD than those that use information of the underlying classifier (i.e. confidence estimation). Yet, most built-in confidence measures of recent powerful classification methods were not yet benchmarked. The goal of this study is twofold: First, various AD measures are benchmarked in order to identify measures that best characterize the probability of misclassification for individual predictions. Since there is an interplay between classification method and AD measure and since not all AD measures can be computed for every classifier, the optimal match between classifier and AD measure is sought. Second, a comparison of novelty detection against confidence estimation for defining the AD is provided. As an aside, the results of this benchmark are also of interest for setting up conformal predictors, which are an alternative to defining the AD of a chemoinformatic classification model [24–27]. An important ingredient of each conformal predictor is a so-called nonconformity score. AD measures can be used for the latter purpose. The better the nonconformity score characterizes the probability of misclassification of an individual prediction, the more efficient will be the resulting conformal predictor. That in turn means that the best AD measure will result in the most efficient conformal predictor. For more details on conformal prediction, the reader is referred to a recent monograph [28].

Random forests (RF), ensembles of feedforward neural networks (NN), support vector machines (SVM), ensembles of boosted classification stumps (MB), k-nearest neighbor classification (k-NN) and linear discriminant analysis (LDA) are evaluated with various AD measures on ten different benchmark data sets. The selection of classifiers is meant to represent a broad variety of well-established classification techniques. Deep neural networks [29] are not covered here. AD measures are computed for independent test sets, simulating future predictions, and are used to compute receiver operator characteristic (ROC) curves. The area under the ROC curve AUC ROC is the primary benchmark criterion to assess how well a particular AD measure can rank predictions from most reliable to least reliable. The paper is organized as follows: In the next section, a brief overview of the employed methods is given. The focus is on the AD measures. Afterwards, the results are reported and discussed. In the following, matrices are given in bold uppercase letters (**A**) while vectors are represented by bold lowercase letters (**a**).

## Methods

### Classification methods, model validation and benchmarking criteria

RF, NN, SVM, MB, k-NN and LDA were run with hyperparameter settings that perform well on average (mostly default parameters) and no hyperparameter optimization was carried out. This may lead to suboptimal models for some data sets but the differences to the optimal models are expected to be small. Moreover, slightly suboptimal models will in general not alter the ranking of the studied AD measures. Since establishing the latter is the ultimate goal of the study, frozen hyperparameters simplify matters here. The exact settings can be found in Additional file 1. Fivefold cross-validation (CV) was used to estimate the prediction error of the classifiers. Since no hyperparameter optimization is done here and thus no model selection is necessary, there is no model selection bias [30–32]. Hence, fivefold CV represents a repetitive partitioning of the data into a training set and an independent test set, which allows estimating the prediction error and derived metrics unbiasedly for a training set size of 4/5th of the data (i.e. the employed training set size in fivefold CV). If the size of the smaller class was less than 40% of the data set size, random undersampling CV (RUS CV) [33, 34] was used to estimate the prediction error to account for class imbalance. Since class imbalance was not severe in most cases, the differences between plain CV and RUS CV are generally small. Additional file 1 provides more details about model validation and the computation of the employed figures of merit. Three performance curves and benchmarking criteria derived thereof were used: ROC curves [35], cumulative accuracy [9, 23], and predictiveness curves [36–39]. For computing each curve, the data are first properly ranked according to the AD measure. Additional file 1 provides detailed information about the performance curves and the necessary specifics for benchmarking novelty scores with ROC curves as well as significance testing of AUC ROC with a permutation test (see also Additional file 2).

### Data sets and molecular descriptors

All studied datasets are publicly available. A summary of their characteristics is shown in Table 1. More detailed information can be obtained from the corresponding references. As can be seen in Table 1 the data sets vary in terms of size and class ratio. For four data sets (MUSK2 [40, 41], QSAR [42, 43], BBB [44], PGP [45]) the previously published descriptors were used. For the remaining six data sets (FXa [46, 47], Liver [48],CYP1A2 [23, 49], hERG [50], Cancer [51], Ames [52]) the provided SMILES were used to calculate two types of molecular descriptors: *MACCS Keys* (166 bit; the frequency of substructures is recorded) [53] and 181 *MOE descriptors* (those that are rotationally and translationally invariant). In addition to MACCS and MOE descriptors, for CYP1A2 the provided E-State descriptors were also used.

**Table 1:** Characteristics of the studied benchmark data sets

	Descriptor type	No. of objects	No. of descriptors	Class ratio (%)
Musk2	Shape/conformation	6598	166	85/15
QSAR	DRAGON	1055	41	34/66
BBB	Chemical/physical properties	325	9	45/55
PGP	Binarized atom pairs	186	1522	42/58
CYP1A2	MACCS/MOE/E-State	7485	166/181/192	46/54
FXa	MACCS/MOE	435	166/181	36/64
Liver	MACCS/MOE	951	166/181	32/68
hERG	MACCS/MOE	561	166/181	62/38
Cancer	MACCS/MOE	7747	166/181	41/59
Ames	MACCS/MOE	6512	166/181	46/54

For descriptor calculation the Chemical Computing Group’s Molecular Operating Environment (MOE) software (Release 2013.08) was used [54]. A list of the 181 MOE descriptors used in this study can be found in the Additional file 3 (Table S13). Except for the MACCS fingerprints and the binarized atom pairs, all descriptors were auto-scaled, i.e. the column mean was subtracted and the mean-centred data were afterwards divided by the standard deviation of that column. Autoscaling was done prior to model building on the entire data matrix. The source of the data as well as the data are provided in the Additional files 4, 5. Those descriptors that were autoscaled prior to the analysis are provided in their autoscaled form. The remaining descriptors are provided as raw data. That means that the data are provided in the way they were used for the respective computations. In addition to the data, the indices for the fivefold CV splits are also provided. For the Ames data set the previously published partitions were used [52]. For the remaining data sets random partitions were generated.

### Applicability domain measures

Sushko et al. [9, 55] introduced the term *distance to model* (DM) as an umbrella term for applicability domain measures. It represents a metric measure that defines the similarity between the training set objects and test set objects (in the validation phase) or future objects (in the productive phase) for a given predictive chemoinformatic model. It is defined to monotonically increase as the (expected) accuracy of the model decreases. While this term is well established, it is somehow misleading since most employed measures are no distances. Hence, the more general umbrella term of applicability domain measure is preferred here. In accord with Sushko et al., larger AD measures indicate a larger error probability for the respective prediction. The AD measure is the basis for defining the AD. Objects with AD values less than a predefined threshold are considered to be inside the AD. The threshold can be found in different ways. One way would be to set the threshold depending on the expected overall accuracy for future predictions (see ‘Cumulative Accuracy’; Additional file 1) [9]. Another way would be to use the 100 − x% quantile of the training set’s AD values as threshold. This would exclude the x% of the most extreme training set objects and future objects that are more extreme than the threshold [9]. A third way would be to limit the expected maximum local error rate, which is defined here as the expected error rate for a given size of the AD value. If the AD measure works efficiently, there will be a relationship between the error rate and the AD value. This relationship can be used to look up the quantile of the AD values which yields a local error rate smaller than a predefined value (see ‘Predictiveness Curves’; Additional file 1). Using a threshold on the AD value to reject predictions that are deemed too uncertain will be referred to as a reject option in accord with the classification literature [56].

#### DA-index (κ, γ, δ)

This measure is based on the k-NN approach. Either the Euclidean distance (*ED*) or one minus Tanimoto similarity (*TD*) was used as distance measures (see also ‘k-Nearest Neighbor’; Additional file 1). The DA-Index comprises three individual measures: ***κ***, ***γ*** and ***δ*** [9, 10]. ***κ*** represents the distance of the future compound to the *k*th-nearest neighbor in the training set. ***γ*** represents the mean distance of a future compound to its *k*-nearest neighbors, while **δ** corresponds to the length of the mean vector from a future compound to its *k*-nearest neighbors. **δ** was introduced to indicate extrapolation since remote objects result in a large mean vector, while well embedded objects show short mean vectors [10]. In this study *k* = 5 was used and either the *ED* or *TD* were used as distance measure. For *ED* the subscript Euc is used while for *TD* Tan is used (e.g. $\kappa_{Euc}$, etc.). The various distance measures are inversely related to the data set density around the future object (short distances reflect high data density). All three measures represent novelty measures.

#### Cosine (cos_α_)

This measure corresponds to the *SCAvg*-*measure* (see [9]). It is defined by the mean cosine similarity coefficient of a future compound to its three nearest training set neighbors [9]. For the sake of comparability five nearest neighbors were used here. The cosine similarity of two objects ${\mathbf{x}}_{a}$ and ${\mathbf{x}}_{b}$ is the inner vector product of the two descriptor vectors divided by the product of their vector lengths:

$$cos\left( {\alpha_{{{\mathbf{x}}_{a} ,{\mathbf{x}}_{b} }} } \right) = \frac{{\mathop \sum \nolimits_{i = 1}^{p} x_{a,i} \cdot x_{b,i} }}{{\sqrt {\mathop \sum \nolimits_{i = 1}^{p} x_{a,i}^{2} \cdot \mathop \sum \nolimits_{k = 1}^{p} x_{b,i}^{2} } }}.$$

It reflects the angle between two vectors starting at the origin extending to the *a*th and *b*th *p*-dimensional object [15]. Cosine ranges between 0 and 1, where a value of 1 indicates perfect similarity. To transform cosine from a similarity measure to an AD measure (i.e. a dissimilarity measure) $1 - { \cos }\left( {\alpha_{{{\mathbf{x}}_{a} ,{\mathbf{x}}_{b} }} } \right)$ was used. Like the aforementioned distance measures, Cosine is a novelty measure.

#### Class probability estimation

The classification error can be minimized if the classifier outputs the class with the largest probability for a particular object ${\mathbf{x}}_{new}$:

$$\hat{c}\left( {{\mathbf{x}}_{new} } \right) = \mathop {\text{argmax}}\limits_{j} \left( {\hat{p}\left( {j|{\mathbf{x}}_{new} } \right)} \right), j \in \left\{ {1, 2} \right\}.$$

$\hat{p}\left( {j|{\mathbf{x}}_{new} } \right)$ is defined as the estimated conditional probability that object ${\mathbf{x}}_{new}$ belongs to the *j*th class given the predictor variables for that object. It depends on the classifier how exactly this posterior probability is estimated. Some classifiers make particular distributional assumptions. LDA does belong to this class of classifiers. The resulting posterior probability $\hat{p}\left( {\hat{c}|{\mathbf{x}}_{new} } \right)$ can directly be used as a built-in confidence measure to define the applicability domain [18]. It is abbreviated as $\hat{p}_{LDA}$ here. Recall that small AD measures indicate reliable predictions. Hence, the error probability $1 - \hat{p}_{LDA}$ would be by definition the respective AD value. In general, estimating conditional class probabilities for various classification techniques is termed probability estimation [57] or class probability estimation in the literature [58, 59]. The latter term will be used throughout this contribution to indicate that the scrutinized AD measure actually estimates conditional class probabilities. The latter have first been used explicitly for defining the AD in [18]. Class probability estimation uses the information of the trained classifier. As a consequence, all AD measures derived from class probability estimates are confidence measures.

#### Class probability estimates using the local vicinity

Some classifiers make no distributional assumptions but use the local vicinity of an object to compute the probability of class membership. k-NN and RF work this way. Let ${\mathcal{N}}_{0}$ be the (indices of the) *k*-nearest objects to ${\mathbf{x}}_{new}$ in the training set. The probability that ${\mathbf{x}}_{new}$ belongs to class *j* is estimated as the fraction of objects of class *j* in ${\mathcal{N}}_{0}$:

$$\hat{p}\left( {j |{\mathbf{x}}_{new} } \right) = \frac{1}{k}\mathop \sum \limits_{{i \in {\mathcal{N}}_0}} {\mathbb{I}}\left( {c_{i} = j} \right),$$

where $c_{i}$ designates the class label of the *i*th object and ${\mathbb{I}} ( {\text{e)}}$ is the indicator function. The estimated class $\hat{c}\left( {{\mathbf{x}}_{new} } \right)$ is the one with the largest class probability. The respective estimated class probability is designated as $\hat{p}_{kNN}$.

The class probability in a decision tree is similarly estimated with the following changes. Now ${\mathcal{N}}_{0}$ represents the (indices of the) *k* training set objects of the terminal leaf ${\mathbf{x}}_{new}$ is assigned to. As opposed to k-NN, *k* may vary here. Yet, decision trees algorithms also assign the fraction of objects of class *j* in ${\mathcal{N}}_{0}$ as a confidence measure $\hat{p}\left( {j |{\mathbf{x}}_{new} } \right)$ for the class membership of object ${\mathbf{x}}_{new}$. Since RF consist of an ensemble of decision trees, $\hat{p}\left( {j |{\mathbf{x}}_{new} } \right)$ is averaged over all $n_{Tree}$ trees in the ensemble:

$$\bar{p}_{j} \left( {{\mathbf{x}}_{new} } \right) = \frac{1}{{n_{Tree} }}\mathop \sum \limits_{i = 1}^{{n_{Tree} }} \hat{p}\left( {j |{\mathbf{x}}_{new} ,Tree_{i} } \right),$$

where $Tree_{i}$ is the *i*th classification tree of the RF ensemble that determines which terminal leaf ${\mathbf{x}}_{new}$ is assigned to. The average over all estimates of the class probabilities $\bar{p}_{j} \left( {{\mathbf{x}}_{new} } \right)$ is called prediction score for class *j* in the language of classification RF (RFC). The individual class probability estimates may also be weighted by the classification accuracy of each single tree and the class prior probability. Again, the estimated class is the one with the largest class probability, the respective class probability estimate is designated as $\bar{p}_{RFC}$. The error probability $1 - \bar{p}_{RFC}$ would give the proper rank order of AD measures. There is a related AD measure that is sometimes used as a confidence measure with classification RF. For predictions, the future object ${\mathbf{x}}_{new}$ is passed down all $n_{Tree}$ members of the ensemble to obtain $n_{Tree}$ class predictions $\left( {\hat{c}_{i} \left( {{\mathbf{x}}_{new} } \right),i = 1, \ldots , n_{Tree} } \right)$. If no class probabilities are computed, class assignment in random forests is simply based on the majority vote of the ensemble members. Let $v_{j} \left( {{\mathbf{x}}_{new} } \right)$ be the fraction of votes for class *j*

$$v_{j} \left( {{\mathbf{x}}_{new} } \right) = \frac{1}{{n_{Tree} }}\mathop \sum \limits_{i = 1}^{{n_{Tree} }} {\mathbb{I}}\left( {j = \hat{c}_{i} \left( {{\mathbf{x}}_{new} } \right)} \right),$$

then the predicted class $\hat{c}_{RFC} \left( {{\mathbf{x}}_{new} } \right)$ of the ensemble is the one that gets the largest fraction of votes. The fraction $v_{j} \left( {{\mathbf{x}}_{new} } \right)$ for class $j = \hat{c}_{RFC} \left( {{\mathbf{x}}_{new} } \right)$ can directly be used as a confidence measure for class membership of object ${\mathbf{x}}_{new}$. It has been termed concordance [9]. $v_{j} \left( {{\mathbf{x}}_{new} } \right)$ can be thought of as a coarse version of $\bar{p}_{j} \left( {{\mathbf{x}}_{new} } \right)$ (just using 0 and 1 for the summands. i.e. $round\left\{ {\hat{p}\left( {j |{\mathbf{x}}_{new} ,Tree_{i} } \right)} \right\}$). Large performance differences between $v_{j} \left( {{\mathbf{x}}_{new} } \right)$ and $\bar{p}_{j} \left( {{\mathbf{x}}_{new} } \right)$ are not to be expected. Since $\bar{p}_{j} \left( {{\mathbf{x}}_{new} } \right)$ is more fine-grained and has a probabilistic interpretation $\bar{p}_{RFC}$ is benchmarked here in favor of ${\hat{{\nu }}}_{RFC} = \hbox{max} \left( {v_{j} \left( {{\mathbf{x}}_{new} } \right)} \right)$ for random forests. In case of multiple boosting the fraction ${\hat{{\nu }}}_{MB}$ will be used as an alternative to the built-in confidence measure derived from the margin of AdaBoost.M1 (see below). In the latter case $n_{Tree}$ is replaced by the respective number of ensemble members.

#### Class probability estimates using regression

Instead of minimizing the 0–1 loss in classification, regression techniques commonly minimize squared error loss. For classification purposes, the regression algorithm does not model a continuous response variable but simply a dichotomous numerical variable that encodes the class labels. In what follows it is assumed that these target values are $y_{i} = 1$ for class 1 and $y_{i} = 0$ for class 2. If squared error loss is minimized with some regression model using the binary y-variable as response, the regression function $\hat{f}\left( {{\mathbf{x}}_{new} } \right)$ estimates class probabilities [60]:

$$\hat{y}\left( {{\mathbf{x}}_{new} } \right) = \hat{f}\left( {{\mathbf{x}}_{new} } \right) = E\left( {1 |{\mathbf{x}}_{new} } \right) = p\left( {1 |{\mathbf{x}}_{new} } \right).$$

Class assignment is based on the rule:

$$\hat{c}\left( {{\mathbf{x}}_{new} } \right) = \left\{ {\begin{array}{*{20}l} 1 \hfill &\quad {if\, \hat{y}\left( {{\mathbf{x}}_{new} } \right) > 1/2} \hfill \\ 2 \hfill &\quad {otherwise} \hfill \\ \end{array} } \right..$$

In practice problems may occur since $\hat{y}\left( {{\mathbf{x}}_{new} } \right)$ need not be bounded to $\left[ {0,1} \right]$ for all regression techniques (e.g. multiple linear regression and associative neural networks). For real-world problems it is important that the regression function approximates the conditional expectation $E(1|{\mathbf{x}}_{new} )$ well. The better it is approximated, the larger will be the utility of the estimated class probabilities as a confidence measure. For many nonparametric regression techniques $\hat{y}\left( {{\mathbf{x}}_{new} } \right)$ estimates $p\left( {1 |{\mathbf{x}}_{new} } \right)$ consistently [57]. These regression techniques estimate class probabilities asymptotically correctly when the sample size tends to infinity. This is, for instance, the case for k-NN regression [61], neural networks trained with squared error loss and error back propagation [62], and regression random forests [57] all of which are included here. Consistency does, unfortunately, not tell anything about the small sample properties of a particular estimator [58]. Yet, these theoretical results show that regression with a dichotomous y-variable may produce good class probability estimates if $E\left( {1 |{\mathbf{x}}_{new} } \right)$ is well approximated with the data at hand. For defining the AD measure, it is again natural to use the error probability $1 - \hat{p}\left( {1 |{\mathbf{x}}_{new} } \right) = 1 - \hat{y}\left( {{\mathbf{x}}_{new} } \right)$ for objects classified as class 1 or $1 - \hat{p}\left( {2 |{\mathbf{x}}_{new} } \right) = \hat{p}\left( {1 |{\mathbf{x}}_{new} } \right) = \hat{y}\left( {{\mathbf{x}}_{new} } \right)$ for objects classified as class 2 (i.e. the smaller error probability is used).

While motivated slightly differently, a quantity termed *CLASS*-*LAG*, which has already been used successfully [9, 23], returns the smaller error probability for a binary classification problem solved by regression modelling:

$$CLASS\hbox{-}LAG\left( {{\mathbf{x}}_{new} } \right) = \hbox{min} \left\{ {\left| {0 - \hat{y}\left( {{\mathbf{x}}_{new} } \right)} \right|,\left| {1 - \hat{y}\left( {{\mathbf{x}}_{new} } \right)} \right|} \right\}.$$

Since $\hat{y}\left( {{\mathbf{x}}_{new} } \right)$ may not be bounded to $\left[ {0, 1} \right]$, the measure is defined also to penalize deviations from the learned target value outside the interval $\left[ {0, 1} \right]$. If $\hat{y}\left( {{\mathbf{x}}_{new} } \right)$ is bounded to $\left[ {0, 1} \right]$ the smaller of the aforementioned error probabilities can simply be used. This was the measure used here, since all of the regression techniques were bounded to $\left[ {0, 1} \right]$. Here, RF with regression trees (RFR), support vector regression (SVR), and regression neural networks (NNR) are used in combination with *CLASS*-*LAG*. As outlined, *CLASS*-*LAG* is essentially derived from the larger class probability estimate. For a unified notation, the latter will be designated as $\bar{p}_{RFR}$, $\hat{p}_{SVR}$, and $\bar{p}_{NNR}$ depending on the base technique used, where $\bar{p}$ indicates that the estimate was derived from an ensemble average. In principle, *CLASS*-*LAG* could also be used with k-NN regression. However, it is easy to show that this would yield identical results than using $\hat{p}_{kNN}$ from classification k-NN (N.B. $CLASS\hbox{-}LAG\left( {{\mathbf{x}}_{new} } \right) = 1 - \hat{p}_{kNN}$ for classification k-NN). With RF in regression and classification mode a similar argument applies since the output of both simply depends on the fraction of major class compounds in the terminal leaf in the considered case. Nevertheless both variants are studied here since regression trees are trained with a different set of default parameters than classification trees. However, the differences between both variants are expected to be small.

#### Class probability estimates from SVM

SVMs classify a new object according to which side of the decision boundary it is located. This information is given by the sign of the so-called decision value. The magnitude of the decision value depends on the object’s distance to the separating hyperplane and is expressed as a multiple of the width of the margin [63]. This distance has no probabilistic meaning but can be calibrated to obtain a class membership probability. While properly calibrated class membership probabilities are favourable for decision making, they are not needed for benchmarking. The employed benchmark criteria solely depend on the rank order of the AD measures (see below) which is not changed through calibration. To illustrate how this calibration works and since calibrated values are easily obtained for SVMs, the procedure is briefly described. So-called Platt scaling is used for this purpose [19]. The scaling procedure uses the decision value as the explanatory variable and the class label ($y_{i } \in \left\{ {0,1} \right\}$) as response variable to fit a one-dimensional logistic regression $\hat{p}\left( {y_{i} = 1| decval\left( {{\mathbf{x}}_{new} } \right), {\hat{\mathbf{w}}}} \right) = sigm\left( {\hat{w}_{0} + \hat{w}_{1} \cdot decval\left( {{\mathbf{x}}_{new} } \right)} \right)$ [64], where $decval\left( {{\mathbf{x}}_{new} } \right)$ represents the decision value for ${\mathbf{x}}_{new}$, $\hat{p}\left( {y_{i} = 1| decval\left( {{\mathbf{x}}_{new} } \right), {\hat{\mathbf{w}}}} \right)$ the estimate of the class membership probability for the class with $y_{i} = 1$, ${\hat{\mathbf{w}}}$ is the parameter vector which is estimated from the training data, and $sigm\left( \eta \right) = 1/\left( {1 + e^{ - \eta } } \right)$ refers to the sigmoid function. The class membership probability for the class with $y_{i} = 0$ equals to $1 - \hat{p}\left( {y_{i} = 1| decval\left( {{\mathbf{x}}_{new} } \right), {\hat{\mathbf{w}}}} \right)$. The larger of the two values corresponds to the class membership probability of the predicted class and is referred to as $\hat{p}_{SVC}$ here (SVC: SVMs in classification mode). The class probability estimates were computed using the option “−b” of LIBSVM [65]. By default, the decision values for calibrating the probability estimates are derived from a fivefold cross-validation of the training data set. The translation of $\hat{p}_{SVC}$ into an AD measure would again be the error probability  $1 - \hat{p}_{SVC}$.

#### Class probability estimates from classification neural networks

Classification neural networks (NNC) had two output nodes here. Objective function and output function (softmax) assured that the classification neural networks output estimates of the class probability bounded to $\left[ {0, 1} \right]$. The larger of the outputs determines the predicted class. Recall, that a five-membered ensemble was used. The average of the larger outputs is designated as $\bar{p}_{NNC}$. The respective AD measure is again the error probability $1 - \bar{p}_{NNC}$.

#### Confidence measure and class probability estimates from boosting

AdaBoost.M1 assigns the class label based on the sign of the decision function $H\left( {{\mathbf{x}}_{new} } \right)$ as follows [66, 67]:

$$H\left( {{\mathbf{x}}_{new} } \right) = sign\left( {F\left( {{\mathbf{x}}_{new} } \right)} \right) = sign\left( {\mathop \sum \limits_{i = 1}^{{n_{Boost} }} \alpha_{i} \cdot h_{i} \left( {{\mathbf{x}}_{new} } \right)} \right),$$

where $n_{Boost}$ is the number of boosting iterations, $h_{i}$ is the output of the base classifier with $h_{i } \in \left\{ { - 1, + 1} \right\}$ and $\alpha_{i}$ is a weighting factor, which depends on the weighted error rate of the respective ensemble member. For obtaining a confidence measure, it is convenient to normalize the weights so that they sum up to one:

$$\tilde{\alpha }_{i} = \frac{{\alpha_{i} }}{{\mathop \sum \nolimits_{i}^{{n_{Boost} }} \alpha_{i} }}.$$

Normalizing $F\left( {{\mathbf{x}}_{new} } \right)$ gives $f\left( {{\mathbf{x}}_{new} } \right)$ which would not change the class assignment:

$$f\left( {{\mathbf{x}}_{new} } \right) = \mathop \sum \limits_{i = 1}^{{n_{Boost} }} \tilde{\alpha }_{i} \cdot h_{i} \left( {{\mathbf{x}}_{new} } \right).$$

Owing to the normalization, it follows that $f$ has range $\left[ { - 1, + 1} \right]$. $\left| {f\left( {{\mathbf{x}}_{new} } \right)} \right|$ represents the absolute margin of the boosted classifiers where the actual normalized margin is defined as $y_{new} \cdot f\left( {{\mathbf{x}}_{new} } \right)$, where $y_{i} \in \left\{ { - 1, + 1} \right\}$. It can be thought of as a weighted majority vote where each single vote $h_{i} \left( {{\mathbf{x}}_{new} } \right)$ is given weight $\tilde{\alpha }_{i}$ [66]. $f\left( {{\mathbf{x}}_{new} } \right)$ represents the difference between the weight of the base classifiers predicting label −1 and those predicting the alternative label +1. If the predicted label $H\left( {{\mathbf{x}}_{new} } \right)$ is based on a narrow majority (i.e. if $f\left( {{\mathbf{x}}_{new} } \right)$ is close to zero), then the confidence in the prediction is low while an absolute value close to one indicates a high confidence in the prediction [66]. Since boosting was combined with bagging here (MB), the final confidence score was computed as the mean of the ensemble as follows:

$$\bar{f}_{MB} = \frac{1}{{n_{Bag} }}\mathop \sum \limits_{i = 1}^{{n_{Bag} }} f_{i} \left( {{\mathbf{x}}_{new} } \right),$$

where $n_{Bag}$ is the number of bootstrap samples drawn, $f_{i} \left( {{\mathbf{x}}_{new} } \right)$ represents the confidence measure of the boosted decision stump on the *i*th bootstrap sample. To translate $\bar{f}_{MB}$ into an AD measure $1 - \left| {\bar{f}_{MB} } \right|$ could be used. Please recall that in addition to $\bar{f}_{MB}$, the fraction of votes $\hat{\nu }_{MB}$ was also used as a confidence measure for multiple boosting. Under certain assumptions [67, 68], it can be shown that the unnormalized $F\left( {{\mathbf{x}}_{new} } \right)$ can be converted to estimated class probabilities using a similar function as with SVMs:

$$\hat{p}\left( {1|{\mathbf{x}}_{new} } \right) = \frac{1}{{1 + e^{{ - 2 \cdot F\left( {{\mathbf{x}}_{new} } \right)}} }}.$$

The assumptions have been criticized as “dubious” [59, 67]. However, this shows that $F\left( {{\mathbf{x}}_{new} } \right)$ and $f\left( {{\mathbf{x}}_{new} } \right)$ are also related to class probability estimates. Yet, the latter may not be well calibrated owing to the violation of the underlying assumptions. For computing the ROC curve or any other performance plot, it does not matter which of the three measures is used since all transformations between them are monotone and do not change the ranking of the objects.

#### Standard deviation (STD)

The standard deviation $$\hat{\sigma }$$ of quantitative predictions of an ensemble was found to correlate with prediction accuracy [55, 69–71]. Largely varying predictions of an ensemble for a particular compound are expected to be less reliable than those with little variation [9, 22]. The standard deviation *STD* was computed from the output of the ensemble members of regression RF ($$STD_{RFR} )$$ and regression neural networks ($$STD_{NNR} )$$. *STD* belongs to the category of confidence measures.

#### PROB-STD

This AD measure was introduced by Sushko et al. [23] and combines *CLASS*-*LAG* and *STD* into one single AD measure. Consider the prediction $\hat{y}\left( {{\mathbf{x}}_{new} } \right)$ with the standard deviation $\hat{\sigma }$ for object ${\mathbf{x}}_{new}$ which is the output of some regression method using an ensemble. Then *PROB*-*STD* is the area under the normal distribution probability density function (PDF) centred at $\hat{y}\left( {{\mathbf{x}}_{new} } \right)$ with the standard deviation $\hat{\sigma }$ from −∞ to 0.5 (decision value) if class 1 is predicted (i.e., $\hat{y}\left( {{\mathbf{x}}_{new} } \right) > 0.5$) and from 0.5 to +∞ if class 2 was predicted. Put differently, *PROB*-*STD* corresponds to the area under the normal distribution PDF beyond the decision value for the alternative class and thus it characterizes the uncertainty of the prediction. If the prediction is close to the numerical target of one class and the standard deviation has a small value, the *PROB*-*STD* value will be small and indicates a reliable prediction. If the predicted value moves closer to the decision value, the *PROB*-*STD* will increase which indicates a less reliable prediction [9, 23]. For a given distance of the predicted value to the decision value, *PROB*-*STD* will increase stronger for larger standard deviations. The *PROB*-*STD* measure is calculated according to the equation:

$$PROB\hbox{-}STD\left( {{\mathbf{x}}_{new} } \right) = \hbox{min} \left\{ {\mathop \int \limits_{ - \infty }^{0.5} {\text{N}}({\text{z}}|\hat{y}\left( {{\mathbf{x}}_{new} } \right),\hat{\sigma }){\text{dz}},\mathop \int \limits_{0.5}^{ + \infty } {\text{N}}({\text{z}}|\hat{y}\left( {{\mathbf{x}}_{new} } \right),\hat{\sigma }){\text{dz}}} \right\},$$

where $${\text{N}}\left( {z\left| {\hat{y}} \right.\left( {{\mathbf{x}}_{new} } \right),\hat{\sigma }} \right)$$ corresponds to the normal probability density function at value z with mean $$\hat{y}\left( {{\mathbf{x}}_{new} } \right)$$ and standard deviation $$\hat{\sigma }$$. *PROB*-*STD* was computed from the output of the ensemble members of regression RF ($$PROBSTD_{RFR} )$$ and regression neural networks ($$PROBSTD_{NNR} )$$. Like class probability estimates and *STD*, *PROB*-*STD* also belongs to the confidence measures.

## Results

The aim of this study is to systematically evaluate different measures for defining the AD of classification models to identify those that correlate best with the error probability of an individual prediction. Six classification techniques RF, NN, SVM, MB, k-NN, and LDA are evaluated in combination with various AD measures in order to rank these measures for every classification method and to identify matching pairs that perform best. Additionally, it is studied whether confidence or novelty measures are more effective to distinguish reliable from less reliable predictions.

Ten benchmark data sets are analyzed in this study. The previously published descriptors were used for MUSK2, QSAR, BBB and PGP, while for the remaining data sets MACCS keys (166 bit; frequency of substructures) were used as structure descriptors in the following. The primary benchmark criterion for the success of the AD measure is the area under the ROC curve (AUC ROC). In addition to that, all accuracy, sensitivity and specificity values for all data sets, studied CV variants and available descriptors can be found in Additional file 3: Tables S1–S10. AUC ROC characterizes the ability of a (classifier-generated) measure to produce a good ranking of class membership for each object [35]. Hence, it can be used to assess how well the AD measure separates reliable from unreliable predictions (the reliable predictions for the first class should rank high, etc.). As opposed to cumulative accuracy and predictiveness curves, a ROC curve is independent of the a priori probabilities of the two classes for classifiers that produce a class membership score [35], which is the reason why it is primarily used here.

Table 2 shows AUC ROC for all combinations of classification techniques and AD measures for all ten data sets. To avoid overinterpretation of differences in light of the prevalent uncertainty and variability, the AUC ROC values were rounded to two significant digits. Techniques that show the same (rounded) AUC value are considered to be equally good. The data are grouped by classification technique where regression and classification mode for a particular technique were grouped together (e.g. classification and regression RF). Within these groups, all available AD measures were ranked for each data set. Ties were assigned the mean rank. The resulting ranks were averaged across all data sets to obtain a mean rank for the particular AD measure. The AD measures within the groups are sorted according to this mean rank. It is shown in the column before the last one and reflects the overall performance of the AD measure for a given classifier. For each classifier the mean ranks cluster in two groups, with a large gap between them (e.g. mean rank 3.45 vs. 6.10 between *STD* and $\cos_{\alpha }$ for RF). This gap separates confidence measures with overall lower ranks—and thus better performance—from novelty measures. There is not a single case where a novelty measure performs better than a confidence measure. Additionally, each ROC curve was assessed by a permutation test. The number of data sets where the respective AD measure induced a ROC curve significantly different from randomly ranking the individual predictions is given in the last column of Table 2. The respective significant AUC ROC values are printed in bold in Table 2. The same clustering as in the case of the mean ranks can be found here. While confidence measures generally produce rank orders that are significantly different from random rankings, this is often not the case for novelty measures.

**Table 2:** AUC ROC for all classification techniques and AD measures

	MUSK2	QSAR	BBB	PGP	FXa	Liver	hERG	Cancer	Ames	CYP1A2	Mean rank	#Signif.^a^
*RF*												
$\bar{p}_{RFC}$	0.99 ^b^	0.93	0.86	0.85	0.98	0.59	0.86	0.64	0.87	0.90	1.95	10
$PROBSTD_{RFR}$	0.99	0.93	0.85	0.85	0.98	0.59	0.86	0.64	0.86	0.90	2.25	10
$\bar{p}_{RFR}$	0.99	0.93	0.85	0.85	0.97	0.59	0.86	0.64	0.86	0.90	2.60	10
$STD_{RFR}$	0.99	0.93	0.84	0.84	0.98	0.58	0.85	0.63	0.85	0.90	3.45	8
$\cos_{\alpha }$	0.95	0.87	0.82	0.82	0.97	0.56	0.78	0.61	0.81	0.84	6.10	5
$\gamma_{Euc}$	0.95	0.86	0.82	0.81	0.97	0.55	0.79	0.61	0.79	0.85	6.50	4
$\kappa_{Euc}$	0.94	0.86	0.81	0.80	0.97	0.54	0.80	0.61	0.79	0.85	7.10	4
$\delta_{Euc}$	0.94	0.86	0.84	0.79	0.96	0.58	0.78	0.59	0.80	0.82	7.45	1
$\delta_{\text{Tan}}$	0.92	0.85	0.79	0.80	0.95	0.55	0.77	0.60	0.78	0.83	9.10	0
$\gamma_{\text{Tan}}$	0.91	0.85	0.78	0.80	0.94	0.56	0.78	0.58	0.78	0.81	9.70	0
$\kappa_{\text{Tan}}$	0.92	0.85	0.78	0.79	0.94	0.57	0.76	0.59	0.78	0.81	9.80	0
Range	0.08	0.08	0.08	0.06	0.04	0.05	0.10	0.06	0.09	0.09		
*NN*												
$\bar{p}_{NNR}$	1.00	0.92	0.83	0.84	0.98	0.57	0.81	0.62	0.84	0.89	2.00	10
$\bar{p}_{NNC}$	1.00	0.92	0.83	0.84	0.98	0.56	0.82	0.61	0.84	0.89	2.25	9
$PROBSTD_{NNR}$	1.00	0.92	0.82	0.83	0.98	0.57	0.81	0.62	0.84	0.89	2.30	10
$STD_{NNR}$	1.00	0.92	0.79	0.82	0.98	0.56	0.80	0.61	0.83	0.88	3.50	9
$\gamma_{Euc}$	0.99	0.86	0.76	0.82	0.96	0.52	0.77	0.59	0.77	0.85	6.65	4
$\kappa_{Euc}$	0.99	0.86	0.76	0.81	0.96	0.52	0.78	0.59	0.77	0.85	6.75	4
$\cos_{\alpha }$	0.99	0.88	0.76	0.76	0.96	0.53	0.73	0.59	0.79	0.83	7.15	4
$\delta_{\text{Tan}}$	0.97	0.85	0.75	0.81	0.95	0.53	0.76	0.58	0.75	0.82	8.70	2
$\delta_{Euc}$	0.98	0.86	0.78	0.73	0.95	0.54	0.74	0.58	0.77	0.81	8.05	0
$\kappa_{\text{Tan}}$	0.96	0.86	0.76	0.76	0.93	0.55	0.74	0.57	0.75	0.80	9.10	0
$\gamma_{\text{Tan}}$	0.96	0.85	0.75	0.77	0.93	0.55	0.74	0.57	0.75	0.80	9.55	0
Range	0.04	0.07	0.08	0.11	0.05	0.05	0.09	0.05	0.09	0.09		
*SVM*												
$\hat{p}_{SVR}$	1.00	0.90	0.86	0.84	0.97	0.57	0.83	0.61	0.84	0.87	1.50	9
$\hat{p}_{SVC}$	1.00	0.91	0.87	0.82	0.97	0.56	0.83	0.60	0.84	0.88	1.60	9
$\cos_{\alpha }$	0.99	0.87	0.80	0.79	0.95	0.56	0.77	0.59	0.79	0.82	4.30	6
$\gamma_{Euc}$	0.99	0.86	0.82	0.75	0.95	0.54	0.79	0.59	0.78	0.83	4.35	5
$\kappa_{Euc}$	0.99	0.86	0.82	0.74	0.95	0.53	0.79	0.59	0.78	0.83	4.75	5
$\delta_{Euc}$	0.98	0.85	0.83	0.74	0.93	0.56	0.75	0.58	0.77	0.80	5.80	0
$\delta_{\text{Tan}}$	0.98	0.85	0.81	0.74	0.92	0.53	0.76	0.58	0.76	0.80	6.80	0
$\kappa_{\text{Tan}}$	0.97	0.84	0.82	0.72	0.91	0.55	0.75	0.57	0.75	0.78	7.70	0
$\gamma_{\text{Tan}}$	0.97	0.84	0.81	0.72	0.90	0.54	0.75	0.57	0.75	0.78	8.20	0
Range	0.03	0.07	0.07	0.12	0.07	0.04	0.08	0.04	0.09	0.10		
*MB*												
$\bar{f}_{MB}$	0.93	0.90	0.80	0.84	0.97	0.59	0.83	0.58	0.76	0.85	1.20	10
$\hat{v}_{MB}$	0.93	0.89	0.79	0.83	0.97	0.58	0.83	0.58	0.75	0.82	1.80	9
$\cos_{\alpha }$	0.89	0.86	0.75	0.80	0.95	0.57	0.74	0.56	0.74	0.80	4.20	6
$\kappa_{Euc}$	0.90	0.84	0.73	0.73	0.95	0.55	0.77	0.56	0.72	0.80	4.85	4
$\gamma_{Euc}$	0.89	0.84	0.73	0.74	0.95	0.55	0.76	0.56	0.72	0.80	5.10	4
$\delta_{Euc}$	0.87	0.83	0.75	0.74	0.93	0.57	0.77	0.55	0.70	0.75	5.30	1
$\delta_{\text{Tan}}$	0.86	0.82	0.69	0.73	0.92	0.56	0.76	0.55	0.69	0.75	6.90	0
$\gamma_{\text{Tan}}$	0.81	0.79	0.69	0.72	0.89	0.56	0.77	0.54	0.66	0.72	7.80	0
$\kappa_{\text{Tan}}$	0.81	0.79	0.69	0.72	0.89	0.57	0.76	0.54	0.66	0.72	7.85	0
Range	0.12	0.11	0.11	0.12	0.08	0.04	0.09	0.04	0.10	0.13		
*k-NN*												
$\hat{p}_{kNN}$	0.97	0.90	0.83	0.80	0.97	0.57	0.80	0.61	0.82	0.87	1.10	10
$\cos_{\alpha }$	0.94	0.87	0.76	0.79	0.97	0.52	0.78	0.59	0.78	0.83	2.95	7
$\gamma_{Euc}$	0.94	0.85	0.77	0.71	0.97	0.51	0.77	0.60	0.77	0.83	3.40	8
$\kappa_{Euc}$	0.93	0.85	0.76	0.69	0.96	0.51	0.78	0.59	0.77	0.83	4.05	6
$\delta_{Euc}$	0.91	0.82	0.76	0.73	0.94	0.55	0.74	0.58	0.75	0.79	4.70	0
$\delta_{\text{Tan}}$	0.90	0.82	0.74	0.68	0.93	0.53	0.72	0.58	0.74	0.79	5.75	0
$\kappa_{\text{Tan}}$	0.88	0.80	0.73	0.66	0.91	0.56	0.70	0.57	0.72	0.77	6.90	1
$\gamma_{\text{Tan}}$	0.88	0.79	0.73	0.66	0.91	0.55	0.71	0.57	0.72	0.76	7.15	0
Range	0.09	0.11	0.10	0.14	0.06	0.06	0.10	0.04	0.10	0.11		
*LDA*												
$\hat{p}_{LDA}$	0.93	0.90	0.77	0.85	0.97	0.54	0.84	0.58	0.76	0.85	1.10	9
$\cos_{\alpha }$	0.86	0.87	0.75	0.83	0.97	0.52	0.76	0.57	0.71	0.81	3.55	6
$\gamma_{Euc}$	0.86	0.86	0.73	0.79	0.96	0.52	0.79	0.57	0.69	0.82	3.60	6
$\kappa_{Euc}$	0.87	0.85	0.72	0.78	0.96	0.52	0.79	0.56	0.69	0.82	3.90	4
$\delta_{Euc}$	0.84	0.84	0.75	0.78	0.94	0.54	0.77	0.55	0.69	0.78	4.55	0
$\delta_{\text{Tan}}$	0.86	0.83	0.69	0.77	0.93	0.53	0.78	0.55	0.67	0.78	5.55	1
$\kappa_{\text{Tan}}$	0.82	0.81	0.70	0.76	0.92	0.53	0.77	0.54	0.67	0.75	6.75	0
$\gamma_{\text{Tan}}$	0.82	0.81	0.69	0.76	0.92	0.53	0.77	0.54	0.67	0.74	7.00	0
Range	0.11	0.09	0.08	0.09	0.05	0.02	0.08	0.04	0.09	0.11		

^a^Number of data sets where the AD measure performs significantly better than chance based on the 95th percentile (α = 0.05) of the permutation test (see Additional file 1 for a description of the permutation test and Additional file 2 for code of the permutation test)

^b^Underlined values indicate that the AD measure performs significantly better than chance based on the permutation test (for details of the permutation test see also footnote a)

Within the group of novelty measures the best performing AD measure is $\cos_{\alpha }$ followed by $\gamma_{Euc}$ (i.e. the mean distance to 5 nearest neighbors using Euclidean distance). Since the available confidence measures for each classifier vary, a single winner cannot be named. However, the type of confidence measure that constantly ranks first is always the same: it is either the built-in class probability estimate of the respective classification technique or the class probability estimate from the related regression technique. For those classifiers without a regression counterpart (MB, k-NN, LDA), the built-in class probability estimate outperformed all other measures (mainly novelty measures). For those techniques that were run in classification and regression mode, the respective class probability estimates (i.e. $\hat{p}$ and $\bar{p}$) ranked top. In case of RF the classification mode has a slight edge while for NNs and SVMs the regression mode wins. However, the differences with respect to mean AUC ROC (across all data sets), mean rank and the number of significant ROC curves are negligible. The same is true for the ensemble-derived *PROB*-*STD*. It is also in the top ranking cluster for the two ensemble techniques (RF and NN). *STD*, which characterizes the ensemble stability, performs slightly worse.

In Table 2 it can be seen that the differences in AUC ROC between the best and worst AD measure for each data set given a particular classifier range between 0.04 (e.g. RF&FXa) and 0.13 (cf. MB&CYP1A2). The most frequent range is 0.09. The latter range, and thus the impact of the different AD measures, may be considered as rather small. However, the variation for a specific data set is exclusively due to different rankings of the predictions induced by the different AD measures. Please note, that there is a pattern in the ranges. If the classifier performs particularly well (i.e. NN&MUSK2, SVM&MUSK2) or particularly bad (Liver and Cancer data sets), the ranges tend to be small. For those data sets in between these extremes the range of AUC ROC is largest. That means that the impact of the different AD measures depends on the level of difficulty of the classification problem (expressed as AUC ROC) and will be largest for classification problems with intermediate difficulty (range AUC ROC: 0.7–0.9).

The performance of the different AD measures was also studied for different sets of structure descriptors (see Additional file 3: Tables S5–S10) and depending on the employed CV scheme (plain CV vs. RUS CV; see Additional file 3: Tables S1, S2, S5–S7 for slightly imbalanced data sets). Summaries are given in Table S11 for different structure descriptors (CYP1A2) and in Table S12 for the two CV variants (QSAR, hERG). While the actual classification performance sometimes changed, the best performing AD measures always remained the same, namely the built-in class probability estimates.

Thus far, the performance of different AD measures for a given classifier was studied which is the focus here. Next, we take a brief look at classifier performance. The AD measures derived from class probability estimates in classification and regression mode were analyzed. With one exception, where RF&$PROBSTD_{RFR}$ performed better than RF&$\bar{p}_{RFR}$ for the FXa data set, they always performed best. Table 3 shows the mean rank and the mean AUC ROC. The single values for AUC ROC were taken from the respective line of Table 2. For the mean rank the nine different combinations were first ranked for each data set and afterwards the average rank over all data sets was taken, as it was for studying AD measure performance. Since the distribution of AUC ROC values is essentially trimodal, the first and the third quartile are given (there is no pattern in the median owing to this irregular distribution). It can be seen that there are three clusters in the data. The first is made up of the top ranking RFs, the second comprises NNs and SVMs, and the third consists of k-NN, MB and LDA. The same trend can be found in mean AUC ROC and the respective quartiles of AUC ROC.

**Table 3:**
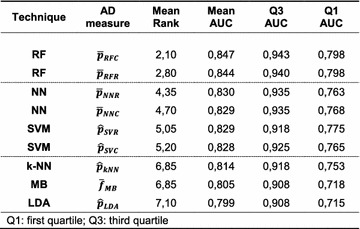
AD measures derived from class probability estimates for all classification techniques

## Discussion

The employed AD measures can be differentiated into novelty measures and confidence measures. Novelty detection seeks to identify novel objects in sparsely populated regions of the data set. Due to lacking near neighbors in the training set, it is assumed that these isolated objects are predicted with less reliability. However, according to the results presented, the data density around a novel object does not very well predict the probability of a prediction error. Basically, two different settings are conceivable: First, the novel object is located on the wrong side of the decision boundary since the latter is not well defined in that region where data are scarce. This is the standard assumption. Second, the novel object is isolated but on the correct side of the decision boundary, e.g. because it is in the tail of the data distribution pointing away from the decision boundary (i.e. it is isolated but far away from the decision boundary). Novelty detectors cannot differentiate these two cases, since they do not use the information of the class labels, they simply flag unusual objects. It follows that objects deemed novel need not show a larger error rate than those that are well embedded in the data. As a consequence, error rate reduction by rejecting the prediction of extreme objects is less efficient using novelty measures as compared to using confidence measures. This does not render novelty detectors useless. Novel objects may be interesting for a number of reasons, e.g. for detecting that novel chemical grounds were hit. However, novelty detection is simply not designed for error rate reduction since it does not use the most valuable resource in that respect: the class labels of the training set objects. Another reason why novelty detection performs worse in this study may be the curse of dimensionality since the employed structure descriptors are rather high-dimensional. Determining distances or data densities is notoriously difficult in high dimensions [72].

If novelty measures are to be used to flag extreme objects, $\cos_{\alpha }$ might be a reasonable choice. It ranked first behind the confidence measures in five out of six cases. Runner-up of the novelty measures was $\gamma_{Euc}$, which is also a reasonable choice. However, it has to be borne in mind that this ranking was determined for AUC ROC und thus with a focus on error rate reduction which may not be the best criterion to assess novelty measures. Recently, specific benchmarks and criteria were studied for assessing the performance of different novelty measures [73, 74]. Interestingly, the novelty measures based on Euclidean distance generally performed better than those using Tanimoto distance. While the differences in mean rank are sizeable, the absolute differences in AUC ROC tend to be small for the two measures. Moreover, most of the rankings according to novelty measures were not significantly different from chance so that it is not possible to draw a definite conclusion about their relative performance.

Confidence measures characterize the distance to the decision boundary. Not unexpectedly, the latter correlates far better with the error probability. Most of the built-in measures directly estimate the class probability (i.e. one minus the error probability). Ranking the data accordingly results in far better AUC ROC values and thus to a more efficient error rate reduction by rejecting the prediction of objects close to the decision boundary (see also below, predictiveness curves). The superior performance of class probability estimates is to be expected from the very purpose of these estimates. The idea of using class probability estimates for rejecting unreliable predictions is everything but new (see [56, 75]) and class probability estimates are in widespread use in other related science fields (see e.g. [57, 76–79]). Yet, a systematic evaluation of these measures for setting the AD in chemoinformatics was still missing. In the aforementioned landmark collaborative study [9], the majority of confidence measures studied here was not included in that benchmark. Moreover, class probability estimates are not broadly applied for setting the AD in chemoinformatics (for exceptions see e.g. [18, 80]). Certainly, conformal prediction, which was recently introduced into chemoinformatics [24], follows a similar philosophy in estimating the reliability of a prediction (by a nonconformity score) for rejecting its prediction if it is too unreliable. As mentioned before, the results obtained here, are also of interest for choosing the nonconformity score. The conformal predictors published thus far in chemoinformatics used confidence measures (either $v_{j} \left( {{\mathbf{x}}_{new} } \right)$ for RFC or $decval\left( {{\mathbf{x}}_{new} } \right)$ for SVC) [24, 25, 27, 81]. While the latter two measures were not explicitly included in the benchmark here, the differences between $v_{j} \left( {{\mathbf{x}}_{new} } \right)$ and $\bar{p}_{RFC}$ are negligible for a reasonably large ensemble of trees and $decval\left( {{\mathbf{x}}_{new} } \right)$ is simply the uncalibrated version of $\hat{p}_{SVC}$ where the calibration does not change the performance of the conformal predictor. The results presented here support this careful choice of the nonconformity score.

Class probability measures can be derived from either classification or regression algorithms. In the two-class case studied here, there are slight differences between classification and regression mode but these are negligible for a practitioner. Hence, it is safe to recommend using the classification mode with the respective class probability estimate. Alternative confidence measures such as *PROB*-*STD* do perform almost as well as the top-ranking class probability estimate and for the practitioner there is little difference for choosing among them. Since the computation of *PROB*-*STD* needs a homo- or hetero-ensemble, it is in many cases more convenient to use the built-in class probability estimate since the latter is computed in any case. It is also of note that *STD*, which characterizes the stability of the ensemble, does not perform as well as the top-ranking class probability estimates. This is noteworthy since *STD* is the measure of choice in regression problems when the reliability of a predicted continuous variable (as opposed to a class probability estimate) shall be assessed [69, 82]. This could be explained as follows: The average output of a regression ensemble is the estimate for the continuous response variable. It is well-known that using the ensemble average typically reduces the prediction error in regression problems. Yet, the ensemble average does not characterize the reliability in regression. Therefore, the standard deviation of the ensemble output is used. In classification, the ensemble average (of the class probability estimate) can directly be used to characterize the reliability of the individual prediction. Using the standard deviation of the ensemble output is not necessary but yields slightly inferior results as compared to the average output of the ensemble (i.e. $\bar{p}$ for RFs and NNs).

**Fig. 1:**
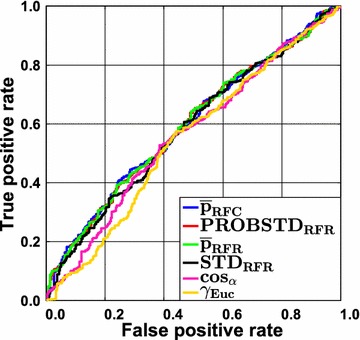
Liver data set employing classification random forests. Receiver operating characteristic (ROC) curves are shown for all confidence measures and the two novelty measures $\cos_{\alpha }$ and $\gamma_{Euc}.$ The overall accuracy is low. Consequently, the differences between the AD measures are rather small (for details see text)

**Fig. 2:**
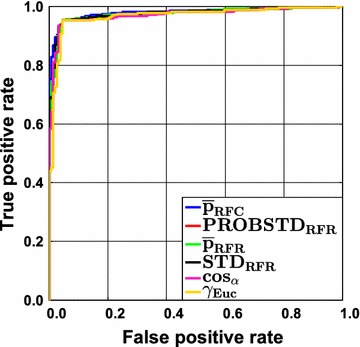
FXa data set employing classification random forests. Receiver operating characteristic (ROC) curves are shown for all confidence measures and the two novelty measures $\cos_{\alpha }$ and $\gamma_{Euc}$. The overall accuracy is extremely high. Consequently, the gain in AUC ROC with the optimal AD measure is limited (for details see text)

The accuracy, and thus AUC ROC, varies across the data sets. Despite this variation, class probability estimates always perform best. However, it has been shown that the gain in AUC ROC depends on the level of difficulty (expressed as AUC ROC). For very difficult classification problems with a high error rate reliable confidence estimation would be most desirable. Yet, in cases where the base classifier does not work well, the class probability estimates are also unreliable. Consequently, the gain in AUC ROC over a random ranking is small. This is illustrated for RF in Fig. 1 for the Liver data set. All ROC curves for this data set are very similar and no notable gain in AUC over a random ranking can be obtained. This shows that it is not possible to enrich the correct predictions at the top and at the end of the ranking list. As a consequence, error rate reduction will rather be negligible when a reject option is employed, even if the best classifier and the best AD measure are chosen. The gain will also be small for very easy classification problems. This is illustrated in Fig. 2 for the FXa data in combination with RF. In this case only few errors occur and the class assignment will be unequivocal in most cases. As a consequence of the low error rate, differences between the ideal ROC curve and a ROC curve with a random ranking will be small. Using simple geometric arguments, it is easy to show that the AUC obtained by randomly ranking the prediction errors (i.e. the median of the permutation distribution) corresponds to $AUC_{random} = 0.5\cdot\left( {Sens + Spec} \right)$ and the best possible AUC would be $AUC_{max} = 1 - \left( {\left( {1 - Sens} \right)\cdot\left( {1 - Spec} \right)} \right)$, where $Sens$ and $Spec$ are the abbreviations for the sensitivity and the specificity of a classifier, respectively. RF using RUS CV results in a sensitivity of 0.953 and a specificity of 0.955 for the FXa data set (Additional file 3: Table S5). In this case even a random ranking of the prediction errors results in an AUC ROC of 0.954, the maximum obtainable AUC ROC would be 0.998. It can be seen that large differences between a random ranking and the ranking according to the optimal class probability estimate will be small. Please recall that we used the 95th percentile of the permutation distribution for assessing the significance of the ranking. That means that a ranking is considered significant only, if it yields a larger AUC ROC than $AUC_{random}$. The actual amount depends on the data set.

**Fig. 3:**
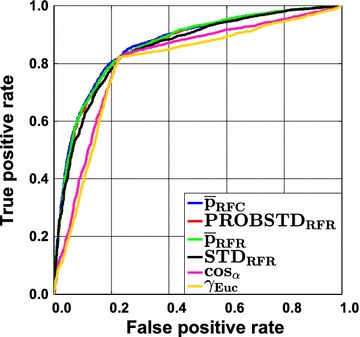
Ames data set employing classification random forests. Receiver operating characteristic (ROC) curves for the classification technique random forest in combination with the Ames data set. The results are shown for the confidence measures and the two novelty measures $\cos_{\alpha }$ and $\gamma_{Euc}$. The inferior performance of the novelty measures can easily be seen. For intermediately difficult problems the impact of a well performing AD measure is largest (for details see text)

**Fig. 4:**
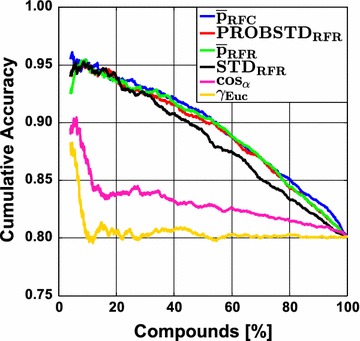
Ames data set employing classification random forests. Cumulative accuracy (CA) curves are shown for all confidence measures and the two novelty measures $\cos_{\alpha }$ and $\gamma_{Euc}$. As with ROC curves, the inferior performance of the novelty measures can easily be seen. CA curves allow reading out the overall accuracy obtained when only a portion of x% of the data is predicted and the rest is rejected

**Fig. 5:**
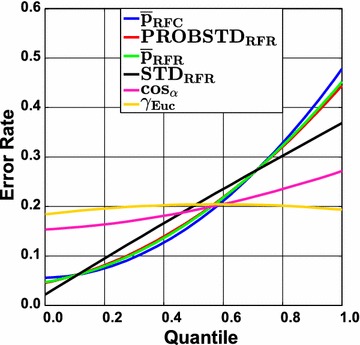
Ames data set employing classification random forests. Predictiveness curves for all confidence measures and the two novelty measures $\cos_{\alpha }$ and $\gamma_{Euc}$ are shown. They show the dependence of the actual error rate depending on the quantile of the AD measure and can be used to set a threshold for the reject option that limits the maximum local error rate

The largest impact of applying the ideal AD measure is expected for intermediately difficult classification problems. This is illustrated in Fig. 3 which shows the RF results for the Ames data set. Here, two clusters of ROC curves can be seen. The set of curves ascending steeper at the beginning belongs to the confidence measures and yield larger AUC ROC values. The curves resulting from novelty measures run more or less linearly from and to the point [$1 - Spec$, $Sens$] which reflects a random ordering of the prediction errors. The AUC ROC values for the different sets of curves vary notably. The class probability estimates are rather reliable in this case and can make a difference as compared to randomly ranking the data. This is corroborated by the $AUC_{random}$ and $AUC_{max}$ values. Random forest classifies the data with a sensitivity of 0.823 and a specificity of 0.770 (see Additional file 3: Table S9) which yields an $AUC_{random}$ of 0.797 and an $AUC_{max}$ of 0.959. The actual AUC ROC of RF&$\bar{p}_{RFC}$ is 0.87. It can be seen that a gain of approximately 0.08 over $AUC_{random}$ can be obtained. Yet, the actual AUC is still away from the ideal one. Since class probability estimates will always be inaccurate in real world applications, it is unrealistic to expect AUC values close to $AUC_{max}$ except for trivial cases. In any case, this example shows that error rate reduction by employing a reject option will have an impact for the intermediate cases. This is illustrated in Figs. 4 and 5 where the cumulative accuracy (CA) and predictiveness curves for RF and the Ames data set are shown. In a cumulative accuracy curve, the accuracy for predictions up to the $\nu$ th quantile of the AD measure is plotted against the quantile $\nu$ (or the percentage of data, respectively). For the CA plots the same two clusters as in the case of ROC curves can be seen. For novelty measures, the CA plot show that only few reliable predictions can be sorted to the top of the ranking list and thus they start lower than the confidence measures and decrease quickly. In a predictiveness curve the error rate associated with the $\nu$th quantile of the AD measure is plotted against the quantile $\nu$. If the AD measure performs well, the error at the beginning of the curve will be small while it should be far larger at the end. A significant slope between the error rate and the AD measure can only be found for the confidence measures, while for the novelty measures the slope is small or insignificant. It can be seen that there is a sharp increase in error rate for the extreme 10–20% of the data when the confidence measures are used as AD measures. For instance, local error rates above 0.3 can largely be avoided if the rejection threshold is set to the 80% quantile of the confidence measures. It can also be seen that the first 30% of the data can be predicted with a local error rate below 0.1 using $\bar{p}_{RFC}$, $\bar{p}_{RFR}$, or $PROBSTD_{RFR}$. In summary, predictiveness curves display the local error rates of the different AD measures, which is well suited to assess the gain that can be obtained with a particular AD measure. While local error rates are very intuitive, no differentiation of FP and FN is possible like in the case of CA curves. This is of particular importance for unbalanced data sets. If no action is taken to re-balance the training of the classifier, predictiveness curves as well as CA plots may be misleading. Since ROC curves are not affected by the class portions it is safer to use them. However, they do not display information about overall or local error rates. Moreover, it has been shown that the differences between $AUC_{random}$ and the AUC of the best performing combination of classifier and AD measure tend to be small which requires careful interpretation of the results. Finally, all three curves allow to reliably identify those AD measures that do not perform better than chance.

## Conclusion

The goal of defining an AD in classification problems is to identify the region in chemical space where “the model makes predictions with a given reliability”. This goal can be achieved in two fundamentally different ways. First, unusual objects can be flagged assuming that they are likely outside the aforementioned region. This was referred to as novelty detection here. Second, unreliable predictions can be flagged which was referred to as confidence estimation. If error rate reduction is the focus of defining an AD, it is mandatory to use confidence measures for defining the AD. Confidence measures will identify objects that are close to the decision boundary and will reject to predict them, which in turn reduces the error rate. From the confidence measures, the built-in class probability estimates performed constantly best, irrespective of the difficulty of the classification problem. Ideal class probability estimates for the studied modelling techniques are listed in Table 3. Alternatives to class probability estimates do not perform better and are inferior in other cases. In the two-class case studied here, differences between learning a classification problem and training a regression algorithm with a dichotomous response variable could not be found. For the sake of simplicity, the general recommendation for efficiently defining the AD would be to train a powerful classifier and use its built-in class probability estimate. In this study random forests once more proved to solve predictive chemoinformatic modelling tasks best. Hence, classification random forests using $\bar{p}_{RFC}$ as built-in confidence measure are a good starting point for defining the AD.

## Additional files

**Additional file 1.** Detailed information about the classification methods, model validation, benchmarking criteria, the comparison between ROC and CA curves and the influence of RUS CV on ROC curves of novelty measures.

**Additional file 2.** Matlab-code for permutation test to determine the distribution of AUC ROC values under the null hypothesis that the AD measure does not carry any information.

**Additional file 3.** Figures of merit (sensitivity, specificity, accuracy, AUC ROC) for all data sets with all CV variants on all descriptors sets (Tables S1–S10). Influence of different descriptor sets for CYP1A2 data set (Table S11). Influence of CV variant for QSAR and hERG data sets (Table S12). MOE descriptors (Table S13).

**Additional file 4.** Source of the data.

**Additional file 5.** Data sets used in this study. The data are provided in the way they were used for the respective computations. In addition to the data, the indices for the fivefold CV are also provided.

## Abbreviations

AD: applicability domain
AUC: area under the curve
AUC ROC: area under the receiver operating characteristic curve
CV: cross-validation
DM: distance to model
k-NN: k-nearest neighbor
LDA: linear discriminant analysis
MB: multiple boosting
MOE: molecular operating environment
NN: neural networks
NNC: classification neural networks
NNR: regression neural networks
PDF: probability density function
Q1: first quartile
Q3: third quartile
QSAR: quantitative structure–activity relationship
RF: random forests
RFC: classification random forests
RFR: regression random forests
ROC: receiver operating characteristic
RUS: random undersampling
SVC: support vector classification
SVM: support vector machines
SVR: support vector regression
