# Supplementary material for: Efficiency of different measures for defining the applicability domain of classification models
Source: J Cheminform. 2017 Aug 3;9:44. doi: 10.1186/s13321-017-0230-2 (PMC5543028; doi:10.1186/s13321-017-0230-2)
Supplement: Supplementary file 1 — Additional file 1. Detailed information about the classification methods, model validation, benchmarking criteria, the comparison between ROC and CA curves and the influence of RUS CV on ROC curves of novelty measures. [file 13321_2017_230_MOESM1_ESM.docx]

**Additional file 1**

**Efficiency of Different Measures for Defining the Applicability Domain of Classification Models**

Waldemar Klingspohn^1^, Miriam Mathea^1^, Antonius ter Laak^2^, Nikolaus Heinrich^2^, Knut Baumann^1^

^1^Institute of Medicinal and Pharmaceutical Chemistry, University of Technology Braunschweig,

Beethovenstrasse 55, 38106 Braunschweig, Germany

^2^Bayer Pharma Aktiengesellschaft, Computational Chemistry, Müllerstrasse 178, 13353 Berlin,

Germany

**Classification Methods**

***Random Forests (RF).*** RF consist of an ensemble of unpruned classification or regression trees [1]. Each tree is built on a bootstrap sample of the training data and at each node the best split is chosen among a randomly selected subset of the predictors. Both perturbation techniques are used to generate a diverse set of ensemble members.

Final predictions are computed as the majority vote of all trees in case of classification or by averaging the individual tree predictions in case of regression [1–3]. All RF calculations were done with the Statistics Toolbox (ver. 9.0) for MATLAB [4]. The ensemble consisted of 300 trees for both, classification and regression analysis. All other parameters were left at their default values. Some important default values are briefly mentioned. The minimum number of observations per tree leaf is set to 1 for classification and it is set to 5 for regression. The number of descriptors to select at random for each node equals to $\sqrt{\text{p}}\text{ }$for classification and to *p*/3 for regression. The prior probability for each class was determined from the class frequencies.

***Neural Networks (NN)*.** Feedforward neural networks are two-stage classification or regression models [5]. They consist of three different layers: an input, a hidden and an output layer [5]. Here, the input layer comprises *p* nodes and the hidden layer was fixed to five nodes. Classification networks had two output nodes since binary classification problems were considered. The output function used for the final transformation of the outputs was the softmax function which assures that the output estimates are positive and sum to one. The objective function was cross-entropy. For regression networks there is a single output node, the output function is the identity function, and the sum of squared errors was minimized. The target value to learn was $y_{i}=1$ for class 1 and $y_{i}=0$ for class 2, respectively. In both cases, the objective function was minimized using backpropagation with weight decay (λ = 0.1) and the employed activation function was the logistic function. With this setup the outputs of the regression network estimate class posterior probabilities (see below) [6]. In order to improve the prediction error, an ensemble with five members was generated by bagging [7–9]. Larger ensembles did not improve the prediction error significantly. Calculations were done with the package nnet (ver.73-12) [10] for R [11]. Except for using weight decay and the hidden layer size, all remaining parameters represent default parameters.

***Support Vector Machines (SVM).***  SVM first project the original data into a high-dimensional or even infinite-dimensional space by using kernel functions. Next a hyperplane is computed that separates the classes to best possible extent. [12–14].

All SVM calculations were computed using the LIBSVM package (ver. 3.17) [15] for MATLAB. The regularization parameter *C* is set to 100 for classification and to 10 for regression. The radial basis function was chosen as the kernel function. The kernel width parameter (γ) was left at its default value 1/*p*. The parameter ε for support vector regression was 0.1 (default). All remaining parameters were also left at their default values.

***Multiple Boosting (MB)*.** Multiple boosting is an ensemble learning technique that combines bagging [9] and boosting [16]. For each bootstrap sample of the training set a base classifier is boosted for $n_{Boost}$ iterations using AdaBoost.M1 [17]. The base classifier chosen here was a one-level decision tree, also called a decision stump [18]. It is a regular decision tree with one root and just two leaves. Boosting a decision stump is repeated for $n_{Bag}$ different bootstrap samples. Each boosted decision stump outputs a class assignment for a future object. The finally predicted class label for the future object is based on the majority vote of the $n_{Bag}$ ensemble members (i.e. $n_{Bag}$ boosted decision stumps). $n_{Bag}$ was set to 100 here and each decision stump was boosted for 10 iterations. All computations were performed by using the Statistics Toolbox (ver. 9.0) for MATLAB.

***k-Nearest Neighbor (k-NN)*.** The k-NN method follows the assumption that similar objects likely belong to the same class [19]. The class assignment of a new compound with unknown class label is determined by the majority class of the *k*-nearest neighbors, i.e. those *k* training set compounds with the smallest distances to the new compound [20–22]. For regression analysis, continuous property values can be predicted as the average property value of the *k*-nearest neighbors. Commonly employed distance measures in cheminformatics are Euclidean distance and Tanimoto similarity [23]. Let the vector $\mathbf{x}$ of dimension *p* x 1 represent the *p* explanatory variables of a chemical compound. The Euclidean distance *ED* between two compounds $\mathbf{x}_{a}$ and $\mathbf{x}_{b}$ can be calculated as follows:

$$ED\left( \mathbf{x}_{a},\mathbf{x}_{b} \right)=\sqrt{\sum_{i=1}^{p} \left( x_{a,i}-x_{b,i} \right)^{2}}.$$

The *Tanimoto* similarity coefficient *TS* for continuous variables is computed as follows:

$$TS\left( \mathbf{x}_{a},\mathbf{x}_{b} \right)=\frac{\sum_{i=1}^{p} x_{a,i}\cdot x_{b,i}}{\sum_{i=1}^{p} x_{a,i}^{2}+\sum_{i=1}^{p} x_{b,i}^{2}-\sum_{i=1}^{p} x_{a,i}\cdot x_{b,i}}.$$

The respective distance measure *TD* is obtained as follows:

$TD\left( \mathbf{x}_{a},\mathbf{x}_{b} \right)=1-TS\left( \mathbf{x}_{a},\mathbf{x}_{b} \right)$*.*

In high-dimensional data spaces (i.e. large *p*) it is rather difficult to differentiate between near and far neighbors. Under certain assumptions, it can be shown that there is a loss of contrast between near and far neighbors as dimensionality increases [7]. This is also known as the “curse of dimensionality” [24]. It has been demonstrated that the Euclidean distance is rather susceptible to the curse of dimensionality [25]. In the virtual screening literature, the Tanimoto similarity coefficient often shows better results than the Euclidean distance [26, 27] which is the reason why it was chosen as sole similarity measure for k-NN classification and regression here.

The size of the neighborhood *k* is a hyperparameter of the method [19], which was set to *k* = 5. All k-NN calculations were computed using the Statistics Toolbox (ver. 9.0) for MATLAB. In case of classification, the prior probability for each class was by default determined from the class frequencies. All remaining parameters were left at their default values.

***Linear Discriminant Analysis (LDA).*** LDA is an early classification technique which due to its simplicity is still widely used [28–30]. In LDA each class is modeled as a multivariate normal distribution with the same covariance matrix $\mathbf{C}$ but a different mean vector ${\bar{\text{x}}}_{\text{j}}$ for class *j*. Without loss of generality only the two-class case (*K* = 2) is considered here, i.e. *j* ∈ {1, 2}. The density $\hat{f}_{j}(\mathbf{x}_{new})$ for the unknown future object $\mathbf{x}_{new}$ given data set $\mathbf{X}$ (*n* x *p*) and class *j* is as follows:

$$\hat{f}_{j}\left( \mathbf{x}_{new} \right)= \frac{1}{\left( 2\pi\right)^{\frac{p}{2}}\cdot\left| \hat{\mathbf{C}} \right|^{\frac{1}{2}}}e^{-\frac{1}{2}\left( \mathbf{x}_{new}-{\bar{\mathbf{x}}}_{j} \right)^{T}{\hat{\mathbf{C}}}^{-1}\left( \mathbf{x}_{new}-{\bar{\mathbf{x}}}_{j} \right)},$$

where $\hat{\mathbf{C}}$ is the pooled sample covariance matrix $\hat{\mathbf{C}}\text{ }\text{=}\text{1}/{\text{(}\text{n}_{\text{1}}\text{+}\text{n}_{\text{2}}-\text{K}\text{)∙(}\text{X}_{\text{1}}^{\text{T}}\mathbf{X}_{1}\text{+}\text{X}_{\text{2}}^{\text{T}}\mathbf{X}_{2}\text{)}}$.

The submatrices $\mathbf{X}_{1}$ (*n*_1_ x *p*) to $\mathbf{X}_{2}$ (*n_2_* x *p*) of $\mathbf{X}$ are composed of the objects of class 1 and 2, respectively, and are assumed to be mean centered with their respective class mean vector ${\bar{\mathbf{x}}}_{j}$.

Let $\pi_{j}$ be the prior probability of class *j* with $\sum_{j=1}^{K} \pi_{j}=1$, then according to Bayes’ Theorem the estimated posterior probability that $\mathbf{x}_{new}$ is of class *j* is:

$$\hat{p}\left( C=j | {X\mathbf{=x}}_{new} \right)= \frac{\hat{f}_{j}(\mathbf{x}_{new})\cdot\pi_{j}}{\sum_{j=1}^{K} \hat{f}_{j}(\mathbf{x}_{new})\cdot\pi_{j}}.$$

Here, $C$ designates a random variable denoting the class and $X$ represents a vector of random variables. Please note that we will mostly use the shorthand $p\left( j|\mathbf{x}_{new} \right)$ or $\hat{p}\left( j|\mathbf{x}_{new} \right)$, respectively. The future compound is assigned to the class with the larger posterior probability [30]. All LDA calculations are performed by using the Statistics Toolbox (ver. 9.0) for MATLAB. The prior probabilities for each class were by default determined from class frequencies. Since the pooled covariance matrix $\left| \hat{\mathbf{C}} \right|$ may become singular and would thus not be invertible, the molecular descriptors were preprocessed by principal component analysis [31] prior to LDA as follows: Let $\mathbf{X}_{c}\boldsymbol{=}\mathbf{X}\boldsymbol{-}\mathbf{1}\boldsymbol{\cdot}\bar{\mathbf{x}}$ be the column mean centred data matrix, let $\mathbf{X}_{\mathrm{AS}}$ be the autoscaled data matrix $\mathbf{X}_{\mathrm{AS}}=\mathbf{X}_{c} . / \left( \mathbf{1}\cdot std\left( \mathbf{X} \right) \right)$, and let $\mathbf{V}$ be the eigenvector matrix of $\text{X}_{\text{AS}}^{\text{T}}\text{·}\text{X}_{\text{AS}}$ sorted according to their eigenvalues, then the scores matrix $\mathbf{T}$ is given as follows: $\mathbf{T}=\mathbf{X}_{\mathrm{AS}}\cdot\mathbf{V}$. Here, the operator ./ designates element-wise division and $std\left( \mathbf{X} \right)$ represents a row vector of the column standard deviations of $\mathbf{X}$. The first 30 columns of $\mathbf{T}$ were used as input for LDA except for the BBB data set where the entire matrix $\mathbf{T}$ was used (i.e. nine columns). Please note that future objects need to be centered with the column mean $\bar{\mathbf{x}}$ and be divided element-wise by $std\left( \mathbf{X} \right)$.

**Model Validation**

The goal of the study is to assess whether or not future compounds fall within the applicability domain (AD) of different classification models. More specifically, different AD measures are benchmarked how well they can differentiate reliable and unreliable predictions for future compounds. Since no real future compounds are available for the studied benchmark data sets, the classifiers are tested by holding out a random subset of the data set compounds from model building. In order to use the data efficiently, the process of partitioning the data into training set and hold-out data set is repeated. In detail, a single *5-fold cross-validation* (CV) is used to simulate future predictions. Here, the data set is randomly divided into five folds (groups) of roughly equal size. The classifier is trained on four folds and the remaining fold is treated as the test set. This is repeated five times. Each time a different fold of objects is treated as the test set. On completion, each object is predicted exactly once. The predictions are stored and used to compute different AD measures and figures of merit. It may be argued that a single 5-fold CV does not properly simulate the prediction of future compounds (often called external validation). However, please note that no hyperparameter optimization is done here and thus no model selection is necessary which in turn avoids model selection bias [32–34]. Hence, the 5-fold CV represents a repetitive partitioning of the data into a training set and an independent test set which allows to estimate the prediction error and derived metrics unbiasedly for a training set size of 4/5th of the data (i.e. the employed training set size in 5-fold CV). The set of hyperparameters, that was selected here, performs well on average. This may lead to suboptimal models for some data sets but the differences to the optimal models are expected to be small. Moreover, slightly suboptimal models will in general not alter the ranking of the studied AD measures. Since establishing the latter is the ultimate goal of the study, frozen hyperparameters simplify matters here.

The following figures of merit were used to characterize classifier and AD measure performance. The predictive performance (*classification accuracy;* $Acc$*)* can be estimated with a hold-out test set which is independent of model building and model selection as follows:

$$Acc=\frac{1}{n_{Test}}\sum_{i=1}^{n_{Test}} \mathbb{I}\left( y_{i}=\hat{y}_{i} \right),$$

where $y_{i}$ is the true class label of the $i$th object, $\hat{y}_{i}$ is the predicted class label of the $i$th object and $\mathbb{I}(e)$ is the indicator function defined as follows:

$$\mathbb{I}\left( e \right)=\left\{ \begin{matrix} 1 \mathrm{if} e is true \\ 0 \mathrm{if} e is false \end{matrix} \right..$$

Hence, the classification accuracy characterizes the fraction of correctly classified test objects. Please note that $n_{Test}=n$ here, since each object is removed from the training set exactly once. The accuracy does not differentiate between the two different error types a binary classifier can produce. This limits its use particularly in unbalanced data sets. Let class one represent the positives and class two represent the negatives. If a positive is misclassified as negative, it is designated as false negative (FN). If a negative is misclassified as positive, it is designated as false positive (FP). Correctly classified objects are referred to as true positives (TP) or true negatives (TN), depending on the respective class label. These four outcomes of a binary classifier form the basis for the performance metrics which differentiate between FN and FP. The *sensitivity (*$Sens$*)*, also called *true positive rate*, *hit rate* or *recall,* gives the fraction of correctly classified positives:

$$Sens=\frac{TP}{\left( TP+FN \right)}.$$

The *specificity (*$Spec$*)*, also called *true negative rate*, gives the fraction of correctly classified negatives:

$$Spec=\frac{TN}{\left( TN+FP \right)}.$$

Related to specificity and useful for computing ROC curves is the so-called *false positive rate* ($FPR$), which characterizes the misclassified negative objects [35]:

$$FPR=1-Spec=\frac{FP}{\left( TN+FP \right)}.$$

***Imbalanced Data Sets*.** Most classifiers work well when the class distribution of the response variable of the data set is balanced. However, data sets for real problems are often imbalanced. There are different approaches to handle imbalanced data sets [36, 37]. In this study resampling is used to reduce the impact of class imbalance. There are two basic sampling techniques. They include random oversampling (ROS) and random undersampling (RUS). In the first technique (ROS) minority class objects are randomly duplicated and in the second technique (RUS) majority class objects are randomly discarded to change the class distributions. Both methods have disadvantages. A drawback of the ROS technique is the possibility of over-fitting, because identical copies of the minority objects are generated. A disadvantage of the RUS technique is that potentially useful majority objects could be discarded [36, 38–40]. In this study the RUS CV technique is compared to plain unmodified CV, because it performed better than the ROS technique in preliminary tests. RUS was implemented as follows: in each CV fold as many objects from the majority class were randomly drawn as there were objects from the minority class. The sampling was carried out in each fold independently from the available training set objects. As a result, the majority class objects changed not only because of the resampling due to CV but also due to the random undersampling. If the class ratio was larger than 60/40, RUS was used by default, although, differences to plain CV sampling were small.

**Benchmarking Criteria**

For computing performance criteria, the objects are first ranked according to their AD measure in ascending order ignoring class information. In the following, it is assumed that objects closest to the training set (i.e. whose predictions are most reliable) are located on the left end of the ranking list and remote objects (i.e. whose predictions are least reliable) are located on the right end of the list (see Figure 1). A perfect AD measure (i.e. best-case scenario) would induce a ranking where all correctly classified objects are ranked before the misclassified objects (see top chart in Figure 1). The better an AD measure induces this perfect ranking, the better it performs. For real data such a perfect ranking cannot be expected. Here, the AD measures of misclassified objects are interspersed among the AD measures of correctly classified objects decreasing the performance of the AD measure (see middle chart in Figure 1). The worst-case scenario would be obtained if the AD measures of misclassified objects are equally distributed among the AD measures of the correctly classified objects (see bottom chart in Figure 1). In this case, the AD measure induces a ranking that is no better than chance (i.e. there is no separation of reliable and unreliable predictions).

In the following three benchmarking criteria (cumulative accuracy, ROC curves and predictiveness curves) are used which are described hereafter.

Fig. 1 Ranking schemes of objects according to their AD measure in ascending order ignoring class information. Top: Ranking of a perfect AD measure where all correctly classified objects are ranked before the misclassified objects. Middle: Real-case scenario. Misclassified objects are interspersed among correctly classified objects. Bottom: Worst-case scenario. No separation of reliable and unreliable predictions (i.e. ranking is no better than chance).

**Cumulative Accuracy (CA).** The so-called cumulative accuracy is based on the aforementioned ranking list according to the AD measure [41, 42]. Based on this ranking, the accuracy for the first *x%* of objects is computed. Next, the accuracy can be computed for the (*x*+1)%, (*x*+2)% and so on up to 100% of objects (i.e. the accuracy is computed for a cumulating portion of the ranking list). The step size need not be 1% and can be chosen to best suit the purpose of the analysis. Finally, all (cumulative) accuracy values are plotted against the respective percentage of included objects (i.e. the quantile of the AD values). The course of this curve characterizes the performance of an AD measure. If the measure performs well, the accuracy for the top-ranked objects (i.e. the most reliable predictions) should be high and will decrease with additional objects included since misclassified objects are more and more likely. The curve will end at the overall accuracy of the underlying classification method (i.e. $Acc$). If the curve starts on the level of the overall accuracy and runs more or less a horizontal on that level, this particular measure cannot separate reliable from unreliable predictions and thus performs poorly. Figure 2 (left chart) illustrates the CA for two hypothetical rank orders.

In case of the blue curve (CA_max_) all correctly classified objects are placed before the misclassified objects. Hence, the accuracy remains 1.0 until the first misclassified object is encountered. Hereafter, the accuracy decreases [continuously](http://www.dict.cc/englisch-deutsch/continuously.html) to the overall accuracy of 0.8. This would represent the perfect AD measure.

**Fig. 2** Cumulative Accuracy (CA) curves (left chart) and Receiver Operating Characteristic (ROC) curves (right chart) for two hypothetical ranked AD measures. Blue curves (CA_max_ and AUC_max_) correspond to the best-case scenario and red curves (CA_random_ and AUC_random_) correspond to the worst-case scenario. (Legend see Figure 1).

In the case of the red curve (CA_random_) misclassified objects are equally distributed among the correctly classified objects. The accuracy remains 0.8. This would represent the worst AD measure that cannot distinguish between reliable and unreliable predictions. Any curve that runs between these limits reflects a varying extent of separation between reliable and unreliable predictions. The more the curve resembles the blue curve (especially at the beginning), the better the discrimination is. Put another way, the larger the area under the curve (AUC), the better performs the respective AD measure. While easy to compute and intuitive to interpret, this benchmarking criterion does not differentiate between false positives and false negatives.

**Receiver Operating Characteristic (ROC).** ROC curves [43] are commonly used to visualize and compare the performance of two-class classifiers. They actually assess the ability of a particular (classifier-generated) score to produce a good ranking to differentiate positive objects from negative ones. A perfect ranking in this sense would be one where all positives rank before the negatives (by convention the positives are ranked first). Each negative object ranked before a positive object diminishes the performance criterion (i.e. the area under the ROC curve; AUC ROC). The earlier a negative appears in the ranking list, the larger are the losses in the performance criterion. The opposite is also true. Since it matters where the errors in the ranking list occur, ROC curves are well-suited to assess how well a particular score separates positives from negatives.

For computing the ROC curve, the data are ranked according to a decreasing degree of class membership for the positive class. Since confidence measures naturally provide class probability estimates, the objects can simply be ranked according to these probability estimates in descending order for the positive class (i.e. $\hat{p}(1|\mathbf{x}_{new})$). In the two-class case this implies that the most reliable predictions for the alternative class are at the end of this list and unreliable predictions range in the middle (i.e. at the borderline of the classes; see Figure 2 right chart). A ROC curve is a step curve in which the true positive rate (*TP rate*; $Sens$) is plotted on the y axis against the false positive rate (*FP rate*; $1-Spec$) on the x axis for increasing portions of the ranked data. The area under the ROC curve is a suitable performance metric for the quality of the ranking list [44]. More precisely, the larger the AUC is, the better is the ranking and thus, the better performs the scrutinized AD measure.

Since novelty measures are one-class classifiers, they do not provide class membership probabilities. This is also true for *STD*. Hence, a slight modification is necessary in order to generate ROC curves for these measures [45]. The foundation of a ROC curve is the ranking with respect to the reliability for one particular class. The same ranking can be obtained for novelty measures by combining the class assignment of the classification model and the novelty measure. Therefore, objects which are predicted as positives are ranked from closest to farthest from the training set (i.e. with decreasing reliability for this class). Next, objects which are predicted as negatives are ranked in reverse order and appended to the first ranking list (i.e. with increasing reliability for this class). Again, unreliable predictions are located at the borderline of the two classes. Since ROC curves for novelty measures depend on the class assignment of the classifier, they always run through the point [$1-Spec$, $Sens$] of the underlying classifier. Hence, the ROC curves for the novelty measures depend on the threshold of the classifier for differentiating between positives and negatives. This is in contrast to confidence measures which solely depend on the relative ranking of positives and negatives without further constraints. However, ROC curves for novelty measures as well as for *STD* also reflect the quality of the ranking.

Figure 2 (right chart) illustrates the ROC curves for two different AD measures for a simulated data set with an accuracy, sensitivity and specificity of 0.8. In the first case the AD measure can distinguish perfectly between reliable and unreliable predictions (blue curve). In the second case the AD measure cannot separate reliable from unreliable predictions (red curve). In this study the main criterion for the performance of an AD measure is the AUC ROC. Preliminary tests showed no significant difference between AUC ROC and the area under the cumulative accuracy curve (AUC CA; regarding the ranking of the AD measures). Yet, AUC ROC is preferred here since it is able to differentiate between FN and FP. It has to be noticed, though, that the AUC ROC depends on the performance of the studied classification technique. The better the performance is, the higher will be the AUC ROC (similar argument applies to CA). Hence, comparisons of different AD measures are only meaningful for a single classification method. If the combination of classification method and AD measure shall be ranked, AUC ROC can also be compared across different classifiers.

In order to analyze whether an AD measure performs significantly better than chance, a permutation test [46] for AUC ROC is applied. Random performance means here that the ROC curve is generated with an AD measure in random order. Hence, the AD measure is randomly permuted here. In order to keep sensitivity and specificity constant, the AD measure for all objects classified as positives are permuted separately from those classified as negative. It should be noted that permuting the AD measure irrespective of class membership would retain the overall accuracy of the underlying classification method *but not* the error distribution of FPs and FNs. 10.000 permutations were generated. The 95^th^ percentile is chosen as the significance level (α = 0.05). For more details of the permutation test see the code in the Additional file 3.

**Predictiveness Curves (PC).** While ROC curves are well established to compare classifiers, they have been criticized for the assessment of risk scores in clinical epidemiology [47], which is analogous to benchmarking AD measures. First, from ROC curves (as well as from CA plots), the local error rate ($ER=1-Acc$) for a given value of the AD measure cannot be read out. Second, it has been argued that an improvement in the predictive ability of an AD measure may not properly be reflected by the change in AUC ROC. Predictiveness curves were suggested to overcome these shortcomings [48–51]. In a predictiveness curve the error rate associated with the $\nu$th quantile of the AD measure is plotted against the quantile $\nu$. Specifically, let the error rate associated with the AD measure *s* be

$$ER\left( s \right)=p\left( Err=1 | S=s \right),$$

where $Err$ is defined as $\mathbb{I}\left( c\neq\hat{c} \right)$, $c$ is the true class label, $\hat{c}$ is the predicted class label and *s* represents the respective AD measure. A plot of $ER\left( s \right)$ against $s$ would not be comparable for differently scaled AD measures, which is the reason why the error rate associated with the $\nu$th quantile of the AD measure $ER(\nu)$ is plotted against the quantile $\nu$.$ER(\nu)$ is defined as follows [49]:

$$ER\left( \nu\right)=p\left( Err=1|S=F^{-1}\left( \nu\right) \right),$$

where $F$ is the empirical cumulative distribution function of the AD measure. The predictiveness curve could be estimated by logistic regression [51] using the AD measure as independent variable and the prediction errors as response. However, we found that a binwise estimation of the error rate combined with smoothing the data gave a better approximation. The estimation procedure is as follows: the data are sorted according to the AD measure as in the case of CA. Next, $n_{Bin}=20$ equally populated bins were formed. The prediction error rate is then computed for each bin and is plotted against $\left( \left( \left( i-1 \right)+i \right)/2 \right)/{n_{Bin}}$ (i.e. the quantile midpoint of the bin), where $i$ is the bin index ($i=1, \ldots,n_{Bin}$). Naturally, the error rate estimates per bin vary due to random fluctuations. Therefore, the values are fitted to a second order polynomial which is displayed. The ideal curve is a step curve that shows a null error rate up the quantile given by $Acc$ and then jumps to 1.0 (i.e. the errors are sorted to the end of the ranking list). The worst case would be a horizontal line at the value $1-Acc$ (the errors are equally distributed on the ranking list). As opposed to CA, where errors are cumulated, the predictiveness curve displays the actual error rate for a particular quantile of the AD measure. Hence, it can easily be used to set a reject option that limits the maximum local error. Assume a maximum local error rate of 0.2 would be acceptable for the application at hand. Then the quantile $\nu$ where the predictiveness curve crosses 0.2 on the y axis can be determined and transformed into the actual value of the AD measure. This value $s$ of the AD measure can then be used as a cut-off value beyond which predictions are rejected because they are deemed too uncertain. The same quantile can then be used to determine the overall accuracy from the CA plot.

**ROC curves vs. CA curves and the influence of RUS CV on ROC curves of novelty measures**

As already mentioned in the main body of the paper AUC ROC characterizes the ability of a (classifier-generated) measure to produce a good ranking of class membership for each object. Hence, it can be used to assess how well the AD measure separates reliable from unreliable predictions (the reliable predictions for the first class should rank high, etc.). As opposed to accuracy, a ROC curve is independent of the a priori probabilities of the two classes for classifiers that produce a class membership score. Hence, ROC curves are particularly useful for assessing unbalanced data sets. This is illustrated in Figures 3 and 4. The top panel of Figure 3 shows the CA plot for MB with its built-in confidence measure $\bar{f}_{MB}$ for the unbalanced data set MUSK2 (class ratio: 85/15). The results for plain CV and RUS CV are shown. While plain CV achieves an overall accuracy of 0.90, RUS CV performs worse and reaches only 0.80. This value can be read off at 100% compounds included (see also Additional file 2, Table S1). This observation misleads to conclude that plain CV produces the better classifier. Matter of fact, in plain CV the larger class is classified with 0.98 specificity while only a sensitivity of 0.44 is achieved for the smaller class. Hence, the classifier basically assigns all objects to the larger class resulting in a rather uninformative classifier. The classifier trained with RUS CV sacrifices specificity (0.77) to enable the recognition of the smaller class with a sensitivity of 0.93. Since the worse specificity for the larger class numerically dominates the accuracy, the latter drops to 0.80. Yet, only this classifier is able to differentiate the two classes. The bottom panel of Figure 3 shows the corresponding ROC curves. Except for small variations (induced by the different CV sampling schemes), both curves coincide. This basically means that a differentiation of the two classes can also be achieved for plain CV by shifting the decision value. ROC curves, as opposed to accuracy-derived measures, do not depend on the actually chosen decision value which comes in handy here. Unfortunately, this is only true for those measures that output the class membership degree. Those measures that do not contain this information (i.e. the novelty measures) depend on the predicted class and thus on the decision value of the classifier. This is shown in Figure 4 for the novelty measure $\kappa_{Euc}$ as compared to $\bar{f}_{MB}$ which does not depend on the decision value (top panel: MB, MUSK2, plain CV). As already mentioned, plain CV does not work well with the default decision value. Figure 4 (bottom panel: MB, MUSK2, RUS CV) shows that RUS CV works far better with the default decision value since it changes class proportions while training the classifier to account for the imbalance. Therefore, RUS CV was always utilized if the larger class consists of more than 60% of the objects.


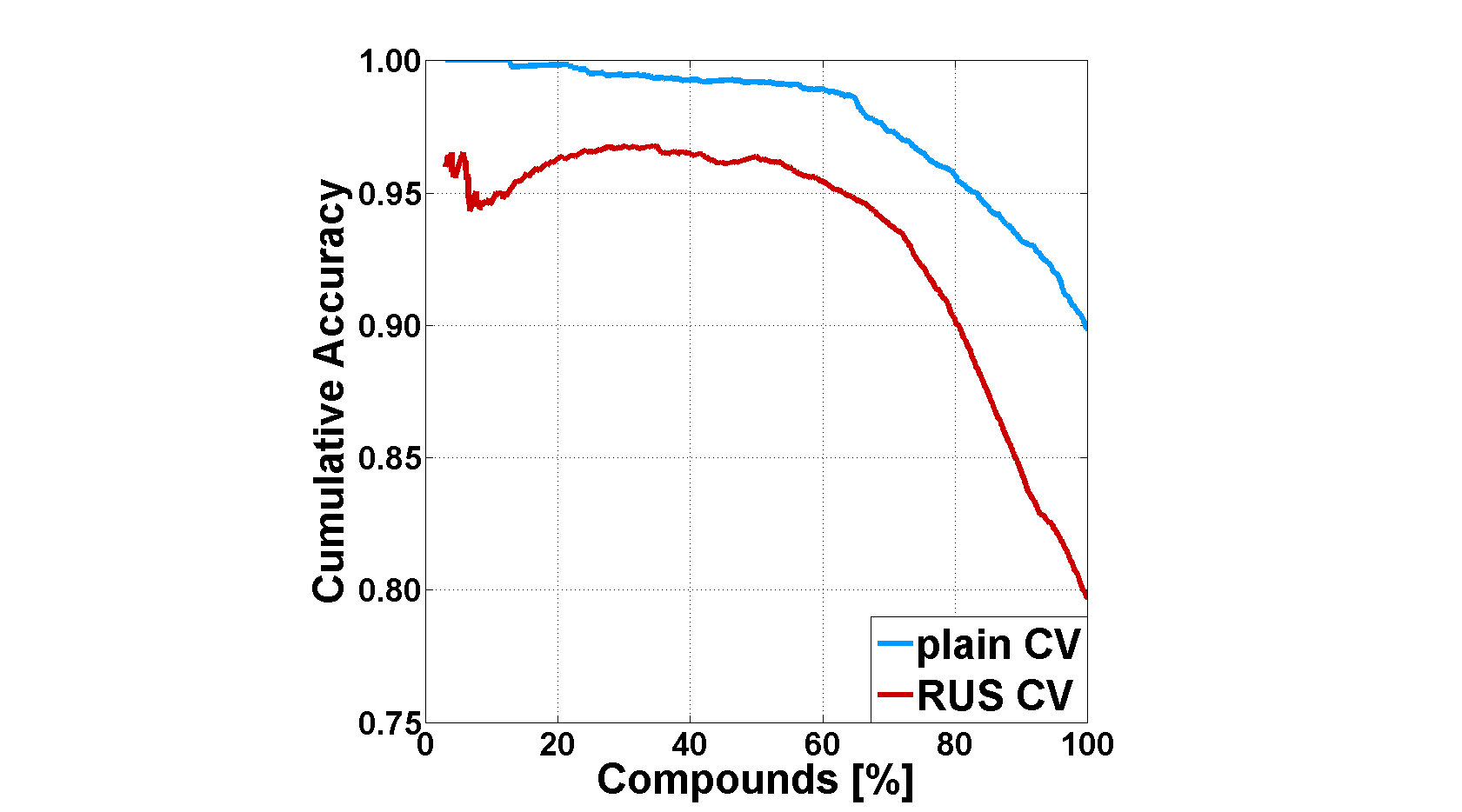

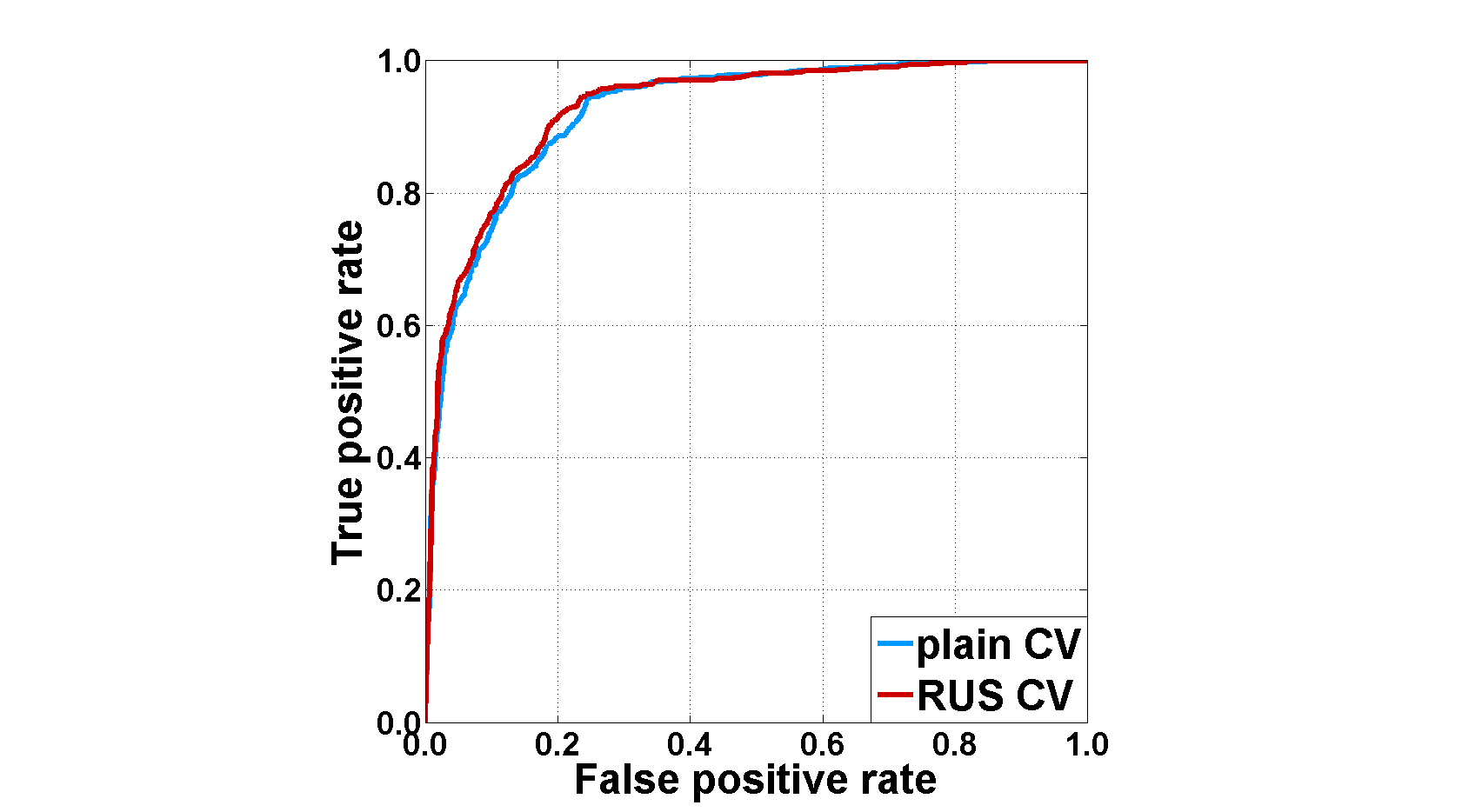


**Fig. 3** Cumulative Accuracy (CA) curves (top panel) and Receiver Operating Characteristic (ROC) curves (bottom panel) for the classification technique multiple boosting with its built-in measure $\bar{f}_{MB}$ for the MUSK2 data set. The results are shown for plain cross-validation (plain CV, blue curves) and random undersampling cross-validation (RUS CV, red curves). Although the overall accuracy of RUS CV is lower, only RUS CV produces meaningful results for the default decision value (for details see text). ROC curves change only marginally.


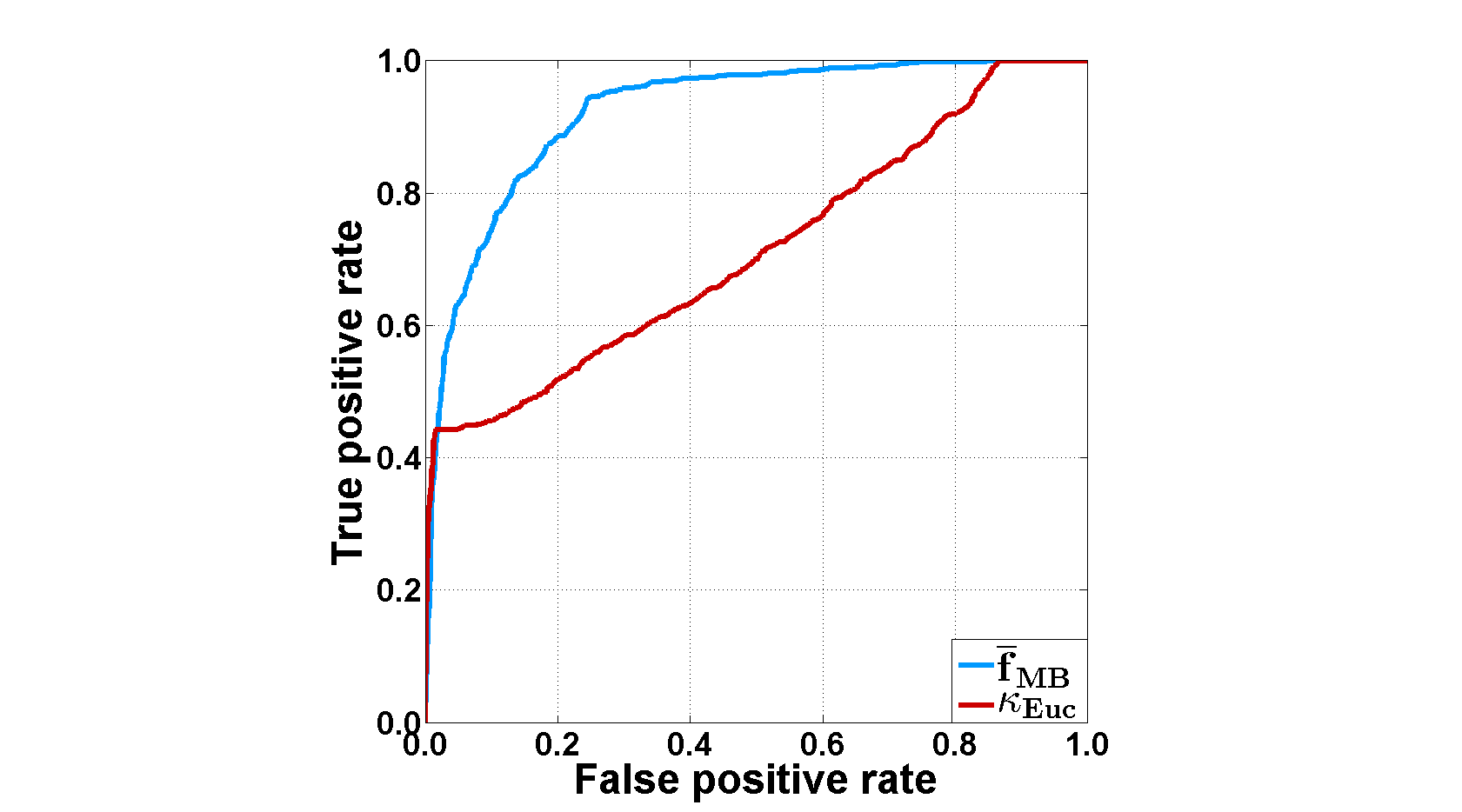

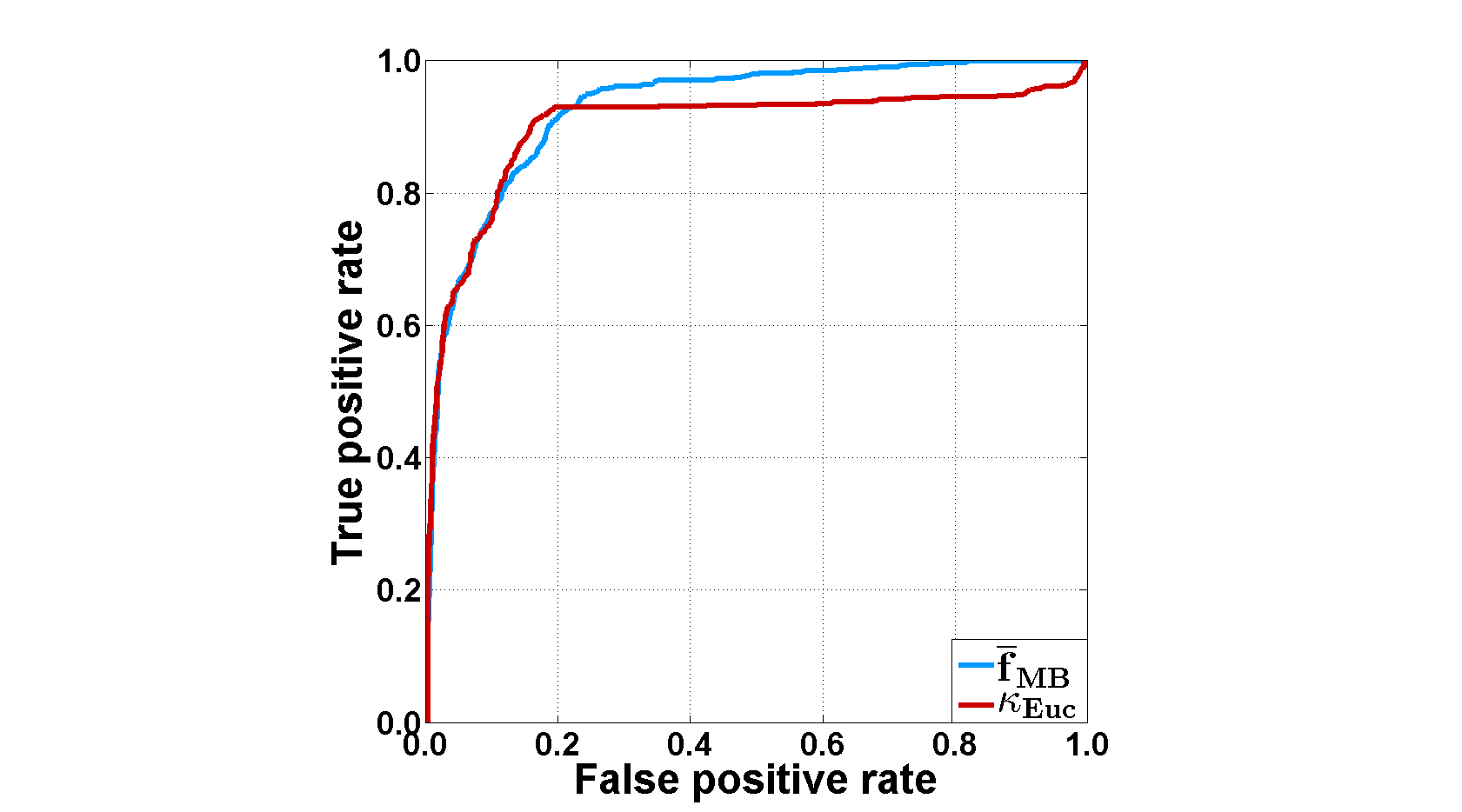


**Fig. 4** Receiver Operating Characteristic (ROC) curves for the classification technique multiple boosting with its built-in measure $\bar{f}_{MB}$ (blue curve) and the novelty measure $\kappa_{Euc}$ (red curve) for the MUSK2 data set. Top: Plain cross-validation. Bottom: Random undersampling cross-validation. While the ROC curves for $\bar{f}_{MB}$ change only marginally, the ROC curve for $\kappa_{Euc}$ is sensitive to class imbalance since it depends on the actual decision value. Only RUS CV yields sensible results with the default decision value (for details see text).

References

1. Breiman L (2001) Random Forests. Mach Learn 45:5–32. doi: 10.1023/A:1010933404324

2. Svetnik V, Liaw A, Tong C, Culberson JC, Sheridan RP, Feuston BP (2003) Random Forest: A Classification and Regression Tool for Compound Classification and QSAR Modeling. J Chem Inf Model 43:1947–1958. doi: 10.1021/ci034160g

3. Svetnik V, Liaw A, Tong C, Wang T (2004) Application of Breiman’s Random Forest to Modeling Structure-Activity Relationships of Pharmaceutical Molecules. In: Roli F, Kittler J, Windeatt T (eds) Multiple Classifier Systems. Springer, Berlin, Heidelberg, pp 334–343

4. MATLAB and Statistics Toolbox Release 2014a, The MathWorks, Inc., Natick, Mass., United States. www.mathworks.com

5. Hastie T, Tibshirani R, Friedman JH (2009) The Elements of Statistical Learning. Data Mining, Inference, and Prediction. Springer, New York

6. Lippmann RP (1989) Pattern classification using neural networks. IEEE Commun Mag 27:47–63. doi: 10.1109/35.41401

7. Mathea M, Klingspohn W, Baumann K (2016) Chemoinformatic Classification Methods and their Applicability Domain. Mol Inf 35:160–180. doi: 10.1002/minf.201501019

8. Rokach L (2010) Pattern Classification Using Ensemble Methods. World Scientific, Singapore

9. Breiman L (1996) Bagging predictors. Mach Learn 24:123–140. doi: 10.1007/BF00058655

10. Venables WN, Ripley BD (2002) Modern applied statistics with S-Plus. Springer, New York

11. The R Project for Statistical Computing: https://www.r-project.org/

12. Burges CJ (1998) A Tutorial on Support Vector Machines for Pattern Recognition. Data Min Knowl Disc 2:121–167. doi: 10.1023/A:1009715923555

13. Müller KR, Mika S, Ratsch G, Tsuda K, Schölkopf B (2001) An introduction to kernel-based learning algorithms. IEEE Trans Neural Netw 12:181–201. doi: 10.1109/72.914517

14. Dudek AZ, Arodz T, Gálvez J (2006) Computational methods in developing quantitative structure-activity relationships (QSAR): a review. Comb Chem High Throughput Screen 9:213–228

15. Chang C-C, Lin C-J (2011) LIBSVM: A library for support vector machines. ACM Trans Intell Syst Technol 2:1–27. doi: 10.1145/1961189.1961199

16. Schapire RE, Freund Y (2012) Boosting. Foundations and Algorithms. MIT Press, Cambridge, Mass.

17. Freund Y, Schapire RE (1997) A Decision-Theoretic Generalization of On-Line Learning and an Application to Boosting. J Comput Syst Sci 55:119–139. doi: 10.1006/jcss.1997.1504

18. Holte R (1993) Very Simple Classification Rules Perform Well on Most Commonly Used Datasets. Mach Learn 11:63–90. doi: 10.1023/A:1022631118932

19. Hand DJ, Mannila H, Smyth P (2001) Principles of Data Mining. MIT Press, Cambridge, Mass.

20. Kauffman GW, Jurs PC (2001) QSAR and k-nearest neighbor classification analysis of selective cyclooxygenase-2 inhibitors using topologically-based numerical descriptors. J Chem Inf Comput Sci 41:1553–1560

21. Ajmani S, Jadhav K, Kulkarni SA (2006) Three-Dimensional QSAR Using the k-Nearest Neighbor Method and Its Interpretation. J Chem Inf Model 46:24–31. doi: 10.1021/ci0501286

22. Zheng W, Tropsha A (2000) Novel Variable Selection Quantitative Structure-Property Relationship Approach Based on the k-Nearest-Neighbor Principle. J Chem Inf Model 40:185–194. doi: 10.1021/ci980033m

23. Willett P, Barnard JM, Downs GM (1998) Chemical Similarity Searching. J. Chem. Inf. Comput. Sci. 38:983–996. doi: 10.1021/ci9800211

24. Aggarwal CC (2001) Re-designing distance functions and distance-based applications for high dimensional data. SIGMOD Record 30:13–18. doi: 10.1145/373626.373638

25. Zimek A, Schubert E, Kriegel H-P (2012) A survey on unsupervised outlier detection in high-dimensional numerical data. Stat Anal Data Mining 5:363–387. doi: 10.1002/sam.11161

26. Todeschini R, Consonni V, Xiang H, Holliday J, Buscema M, Willett P (2012) Similarity coefficients for binary chemoinformatics data: overview and extended comparison using simulated and real data sets. J Chem Inf Model 52:2884–2901. doi: 10.1021/ci300261r

27. Bajusz D, Rácz A, Héberger K (2015) Why is Tanimoto index an appropriate choice for fingerprint-based similarity calculations? J Cheminf 7:1–13. doi: 10.1186/s13321-015-0069-3

28. Fisher R (1936) The Use of Multiple Measurements in Taxonomic Problems. Ann Hum Genet 7:179–188. doi: 10.1111/j.1469-1809.1936.tb02137.x

29. Fukunaga K (1990) Introduction to Statistical Pattern Recognition. Academic Press, Boston

30. Duda RO, Hart PE, Stork DG (2001) Pattern Classification. Wiley, New York

31. Jolliffe IT (2002) Principal Component Analysis. Springer, New York

32. Cawley GC, Talbot NL (2010) On Over-fitting in Model Selection and Subsequent Selection Bias in Performance Evaluation. J Mach Learn Res 11:2079–2107

33. Varma S, Simon R (2006) Bias in error estimation when using cross-validation for model selection. BMC Bioinf 7:91. doi: 10.1186/1471-2105-7-91

34. Baumann D, Baumann K (2014) Reliable estimation of prediction errors for QSAR models under model uncertainty using double cross-validation. J Cheminf 6:47. doi: 10.1186/s13321-014-0047-1

35. James G (2013) An Introduction to Statistical Learning: with Applications in R. Springer, New York

36. Yap BW, Rani KA, Rahman HAA, Fong S, Khairudin Z, Abdullah NN (2014) An Application of Oversampling, Undersampling, Bagging and Boosting in Handling Imbalanced Datasets. In: Herawan T, Deris MM, Abawajy J (eds) Proceedings of the First International Conference on Advanced Data and Information Engineering (DaEng-2013). Springer Singapore, Singapore, pp 13–22

37. Haibo H, Garcia EA (2009) Learning from Imbalanced Data. IEEE Trans Knowl Data Eng 21:1263–1284

38. Chawla NV (2005) Data Mining for Imbalanced Datasets: An Overview. In: Maimon O, Rokach L (eds) Data Mining and Knowledge Discovery Handbook. Springer, New York, pp 853–867

39. Drummond C., Holte R. (2003) C4.5, Class Imbalance, and Cost Sensitivity: Why Under-Sampling beats Over-Sampling. ICML, Washington DC

40. Kotsiantis S, Kanellopoulos D, Pintelas P (2006) Handling imbalanced datasets: A review. GESTS Int Trans Comput Sci Eng 30:25–36

41. Sushko I, Novotarskyi S, Körner R, Pandey AK, Cherkasov A, Li J, Gramatica P, Hansen K, Schroeter T, Müller K-R, Xi L, Liu H, Yao X, Öberg T, Hormozdiari F, Dao P, Sahinalp C, Todeschini R, Polishchuk P, Artemenko A, Kuz’min V, Martin TM, Young DM, Fourches D, Muratov E, Tropsha A, Baskin I, Horvath D, Marcou G, Muller C, Varnek A, Prokopenko VV, Tetko IV (2010) Applicability Domains for Classification Problems: Benchmarking of Distance to Models for Ames Mutagenicity Set. J Chem Inf Model 50:2094–2111. doi: 10.1021/ci100253r

42. Sushko I, Novotarskyi S, Körner R, Pandey AK, Kovalishyn VV, Prokopenko VV, Tetko IV (2010) Applicability domain for in silico models to achieve accuracy of experimental measurements. J Chemometr 24:202–208. doi: 10.1002/cem.1296

43. Fawcett T (2006) An introduction to ROC analysis. Pattern Recogn Lett 27:861–874. doi: 10.1016/j.patrec.2005.10.010

44. Jin H, Ling CX (2005) Using AUC and accuracy in evaluating learning algorithms. IEEE Trans Knowl Data Eng 17:299–310. doi: 10.1109/TKDE.2005.50

45. Gavin DG, Oswald W, Wahl ER, Williams JW (2003) A statistical approach to evaluating distance metrics and analog assignments for pollen records. Quatern Res 60:356–367. doi: 10.1016/S0033-5894(03)00088-7

46. Edgington ES, Onghena P (2007) Randomization tests. Chapman & Hall/CRC, Boca Raton

47. Sullivan Pepe M, Janes HE (2008) Gauging the Performance of SNPs, Biomarkers, and Clinical Factors for Predicting Risk of Breast Cancer. J Natl Cancer Inst 100:978–979. doi: 10.1093/jnci/djn215

48. Copas J (1999) The Effectiveness of Risk Scores: the Logit Rank Plot. J Royal Statistical Soc C 48:165–183. doi: 10.1111/1467-9876.00147

49. Huang Y, Sullivan Pepe M, Feng Z (2007) Evaluating the predictiveness of a continuous marker. Biometrics 63:1181–1188. doi: 10.1111/j.1541-0420.2007.00814.x

50. Sullivan Pepe M, Feng Z, Huang Y, Longton G, Prentice R, Thompson IM, Zheng Y (2008) Integrating the predictiveness of a marker with its performance as a classifier. Am J Epidermiol 167:362–368. doi: 10.1093/aje/kwm305

51. Empereur-mot C, Guillemain H, Latouche A, Zagury J-F, Viallon V, Montes M (2015) Predictiveness curves in virtual screening. J Cheminform 7. doi: 10.1186/s13321-015-0100-8
