# Supplementary material for: Efficiency of different measures for defining the applicability domain of classification models
Source: J Cheminform. 2017 Aug 3;9:44. doi: 10.1186/s13321-017-0230-2 (PMC5543028; doi:10.1186/s13321-017-0230-2)
Supplement: Supplementary file 4 — Additional file 4. Source of the data. [file 13321_2017_230_MOESM4_ESM.docx]

**Additional file 4**

**Efficiency of Different Measures for Defining the Applicability Domain of Classification Models**

Waldemar Klingspohn^1^, Miriam Mathea^1^, Antonius ter Laak^2^, Nikolaus Heinrich^2^, Knut Baumann^1^

^1^Institute of Medicinal and Pharmaceutical Chemistry, University of Technology Braunschweig,

Beethovenstrasse 55, 38106 Braunschweig, Germany

^2^Bayer Pharma Aktiengesellschaft, Computational Chemistry, Müllerstrasse 178, 13353 Berlin,

Germany

**Musk2:**

The data was introduced by the following publication:

Dietterich, TG, Jain A, Lathrop R, Lozano-Perez, T (1994) A comparison of dynamic reposing and tangent distance for drug activity prediction. In: Proceedings of the sixth international conference on neural information processing system, pp 216–223

The data can be downloaded from the following link:

**URL:** <http://sci2s.ugr.es/keel/dataset.php?cod=174>

**QSAR:**

The data can be downloaded from the following link:

**URL:** [http://archive.ics.uci.edu/ml/datasets/QSAR+biodegradation#](http://archive.ics.uci.edu/ml/datasets/QSAR+biodegradation)

Smiles, the descriptors and class labels are also provided in the Supporting Information of the following publication:

**DOI:** 10.1021/ci4000213

**BBB:**

The data can be found in the Appendix of the following publication:

**DOI:** 10.1089/10665270260518317

**PGP:**

The data is provided in the Supporting Information of the following publication:

**DOI:** 10.1021/ci034160g

**CYP1A2:**

The data was introduced by the following publication:

**DOI:** 10.1002/cem.1296

Smiles, class labels and the E-State descriptors can be downloaded from the following link:

**URL:** <https://ochem.eu/models/2>

**FXa:**

The data was introduced by the following publication:

**DOI:** 10.1021/jm049113+

The data can be downloaded from the following link:

**URL:** <http://www.cheminformatics.org/datasets/>

**Liver:**

The data was introduced by the following publication:

**DOI:** 10.1021/tx900326k

Smiles and class labels are provided in the Supporting Information.

**hERG:**

The data was introduced by the following publication:

**DOI:** 10.1021/mp700124e

Smiles and class labels are provided in the Supporting Information.

**Cancer:**

The data was introduced by the following publication:

**DOI:** 10.1021/ci6004004

Smiles and the pGI_50_ values of the 36 cell lines are provided in the Supporting Information.

**Ames:**

The data was introduced by the following publication:

**DOI:** 10.1021/ci900161g

Smiles and the class labels are provided in the Supporting Information as well as the partitions for the 5-fold CV.
